# Statistical indices of masculinity-femininity: A theoretical and practical framework

**DOI:** 10.3758/s13428-024-02369-5

**Published:** 2024-03-04

**Authors:** Marco Del Giudice

**Affiliations:** University of Trieste, Department of Life Sciences, Trieste, Italy

**Keywords:** Gender diagnosticity, Masculinity-femininity, Measurement error, Multivariate analysis, Sex differences

## Abstract

Statistical indices of *masculinity-femininity* (M-F) summarize multivariate profiles of sex-related traits as positions on a single continuum of individual differences, from masculine to feminine. This approach goes back to the early days of sex differences research; however, a systematic discussion of alternative M-F indices (including their meaning, their mutual relations, and their psychometric properties) has been lacking. In this paper I present an integrative theoretical framework for the statistical assessment of masculinity-femininity, and provide practical guidance to researchers who wish to apply these methods to their data. I describe four basic types of M-F indices: *sex-directionality*, *sex-typicality*, *sex-probability*, and *sex-centrality*. I examine their similarities and differences in detail, and consider alternative ways of computing them. Next, I discuss the impact of measurement error on the validity of these indices, and outline some potential remedies. Finally, I illustrate the concepts presented in the paper with a selection of real-world datasets on body morphology, brain morphology, and personality. An R function is available to easily calculate multiple M-F indices from empirical data (with or without correction for measurement error) and draw summary plots of their individual and joint distributions.

**Supplementary information:**

The online version contains supplementary material available at 10.3758/s13428-024-02369-5.

In their seminal book *Sex and Personality*, published almost 90 years ago, Terman and Miles (1936) proposed that individual differences in sex-related traits could be described as positions on a continuum of *masculinity-femininity* (M-F), and measured by statistically combining multiple variables into a single index. By relating them to the corresponding trait distributions in males and females considered as groups, individual profiles can be rated as more or less “masculine” or “feminine,” enabling fine-grained analyses both between and within the sexes. The notion of a bipolar M-F continuum waxed and waned in popularity throughout the twentieth century (see Lippa, 2001); it then experienced a renaissance with the introduction of *gender diagnosticity* (GD; Lippa, 1991; Lippa & Connelly, 1990), a method that employs discriminant analysis to estimate a person’s probability of being male versus female (more on this below). In recent years, researchers have increasingly used GD and other kinds of M-F indices to investigate a variety of topics related to gender and sexuality (e.g., Ilmarinen et al., 2023; Lippa, 2005; Loehlin et al., 2005; Lönnqvist & Ilmarinen, 2021; Pozzebon et al., 2015; Rieger & Savin-Williams, 2012; Semenyna & Vasey, 2016; Udry & Chantala, 2004; Verweij et al. 2016).

The idea of using statistical procedures to calculate continuous M-F scores has some obviously attractive features, including parsimony (complex multivariate profiles are summarized by a single dimension of variation) and flexibility (there is no need to rely on a particular assessment instrument, questionnaire or otherwise). At the same time, treating masculinity-femininity as a statistical construct leaves it open-ended in two important ways. To begin with, the same index may be calculated from different domains of sex-related variation. For example, gender diagnosticity is usually estimated from profiles of occupational preferences, interests, and everyday activities (see Lippa, 2001, 2010), but some authors have used variations on this method to obtain separate GD scores from personality scales, personal values, cognitive abilities, and so forth (Ilmarinen et al., 2023). Empirically, M-F indices calculated over different domains show only small to moderate correlations with one another, indicating that variation in psychological masculinity-femininity is not characterized by a strong underlying “general factor” (Ilmarinen et al., 2023; Pozzebon et al., 2015).

Second, and key to the present paper, there is more than one way to translate individual trait profiles into meaningful M-F scores. The general construct of masculinity-femininity can be conceptualized in a number of different ways, yielding alternative types of indices with their unique properties and implications. Conversely, different methods that are employed to construct M-F scores often embody alternative conceptions of masculinity-femininity (e.g., the extremity of the sex-related traits displayed by an individual, versus the degree to which an individual is statistically representative of males/females as groups). To the best of my knowledge, this point has never been addressed systematically in the psychological literature. The outcomes include not just conceptual and statistical muddles, but also a failure to take advantage of the sophistication and descriptive richness afforded by multiple, complementary indices.

Here I set out to correct this blind spot and provide an integrative framework for the statistical assessment of masculinity-femininity. I begin by describing four basic types of M-F indices, which I label *sex-directionality* (M-F_D_), *sex-typicality* (M-F_T_), *sex-probability* (M-F_P_), and *sex-centrality* (M-F_C_). Each captures a somewhat distinct aspect of the broader construct of masculinity-femininity. It is especially noteworthy that, under certain conditions, the relative ranking of two people’s trait profiles (i.e., which one is more masculine vs. feminine) may switch depending on the index that one is employing. In fact, the *same* profile may lie on the masculine side of the continuum according to one type of index, but on the feminine side according to another. I then examine the differences and relations between alternative indices, explain how they are affected by measurement error, and consider potential remedies. Finally, I illustrate the concepts and methods discussed in the paper with a selection of real-world datasets on body morphology, brain morphology, and personality. These empirical examples offer useful insight into the behavior of alternative M-F indices in different scenarios, their dependence on the distribution of the data, and their sensitivity to measurement error. To facilitate research applications, I provide an easy-to-use R function (*mf.indices*) that calculates multiple M-F indices from empirical data and draws summary plots of their individual and joint distributions. The function can be downloaded at 10.6084/m9.figshare.22277743

Before I begin, I want to stress that my goal is not to defend the superiority of bipolar indices, or discuss their intrinsic limitations in any detail. The contrast between bipolar conceptions of M-F and alternative models that view masculinity and femininity as distinct, at least partly independent dimensions of variation is the subject of a long and still ongoing debate (see Lippa, 2001). I take it for granted that the statistical M-F indices I discuss in this paper are no more than convenient, broad-band summaries, which cannot be expected to capture *everything* of importance about sex-related patterns of individual differences (Del Giudice, 2021; for a recent example in the field of face perception, see Hester et al., 2021). Whether bipolar M-F indices, unipolar M and F measures, or still other approaches are most relevant and informative with respect to a given research question is a complex methodological question that lies beyond the scope of this paper. I believe that M-F indices remain valuable tools in the ever-expanding toolbox of sex/gender research, and that a deeper understanding of their functioning can only help scientists make better, more informed decisions.

## Four types of M-F indices

### Sex-directionality

The first and arguably simplest approach to masculinity-femininity is to conceptualize it as a summary measure of the expression of sexually dimorphic traits. A person is more masculine (or feminine) than another to the extent that his/her trait values are shifted in the male (or female) direction, as defined by the pattern of mean differences between the sexes. Thus, if men are taller than women on average, a taller person will be rated as more physically masculine than a shorter one (all else being equal). And if men have broader shoulders than women on average, a person with narrower shoulders will be rated as more physically feminine than one with broader shoulders (again, all else being equal). Importantly, each trait makes an independent contribution to M-F scores, irrespective of its correlations with the other traits. I propose *sex-directionality* (M-F_D_) as a descriptive label for indices that fit this definition.

Indices of sex-directionality have a long history in psychology. Indeed, the original “M-F test” developed by Terman and Miles (1936) yielded sex-directionality scores, obtained from the sum of “masculine” versus “feminine” responses to a wide assortment of items. Many classic M-F scales—such as the one contained in Strong’s vocational test (Strong, 1943)—were based on the same principle. Likewise, Lippa (1991) contrasted his newly developed GD index with a “traditional” M-F scale built by summing all the items that showed significant sex differences. A recent example is the study by Pozzebon et al. (2015), in which M-F factors for personality, vocational interest, and sexual fantasy were obtained by factor-analyzing various scales selected for their patterns of mean sex differences in previous research.

**Fig. 1:**
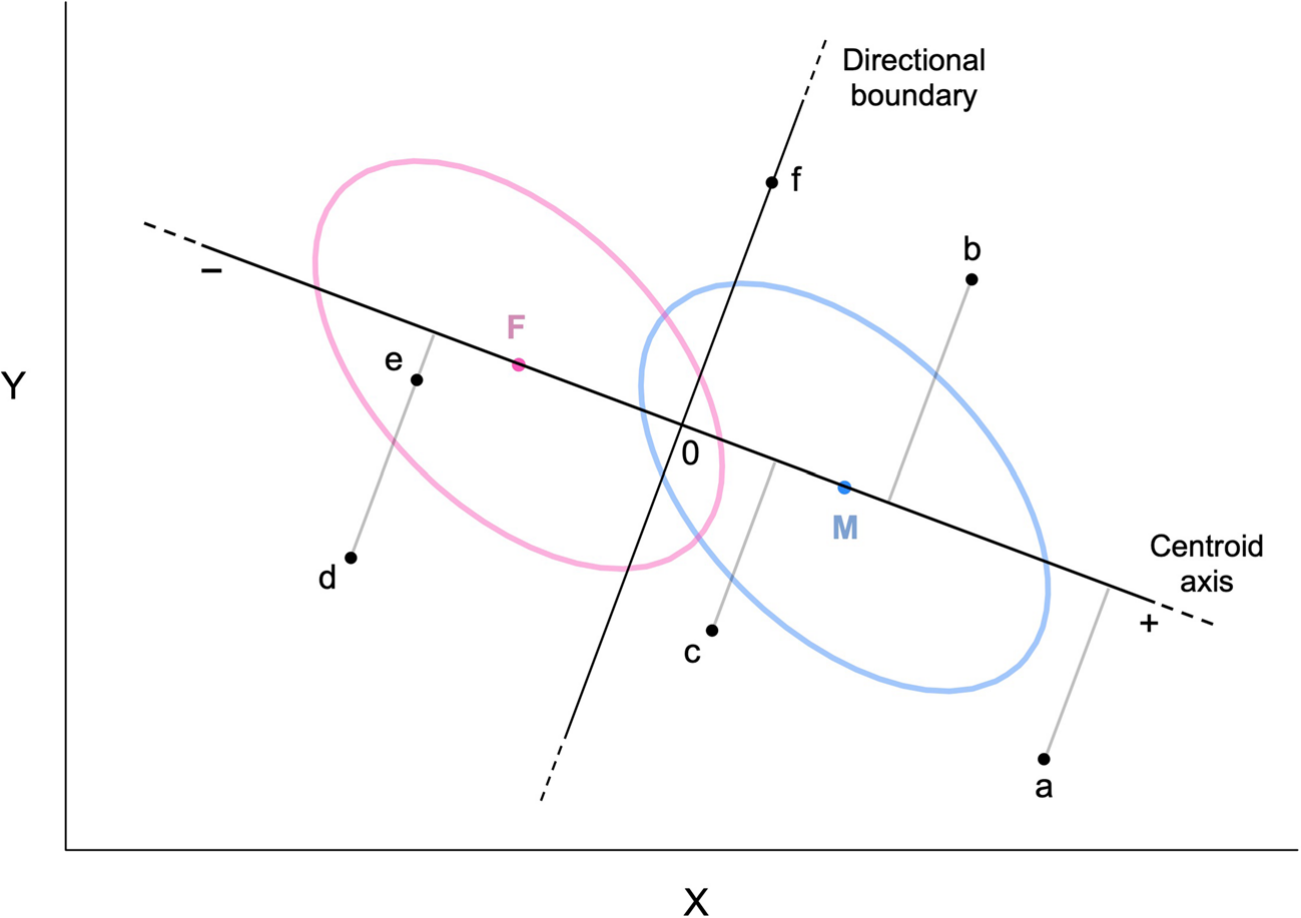
Schematic illustration of sex-directionality with two correlated traits X and Y. Points *M* and *F* are the centroids of the male and female distributions. The bivariate *SD*s of the distributions are shown as ellipses (note: X and Y are assumed to be multivariate normal with equal covariance matrices in the two sexes)

A more precise and rigorous way to measure sex-directionality is to find the linear combination of traits that maximizes sexual dimorphism—what Mitteroecker et al. (2015) referred to as “maleness-femaleness” in relation to the morphological features of human faces. This approach generalizes to other trait domains. Figure 1 presents a simple example involving two negatively correlated traits X and Y, assumed to be normally distributed with equal variances/covariances in the two sexes. Individual trait profiles are represented by points on the plane. *M* and *F* are the centroids (multivariate means) of the male and female distributions; on average, males score higher than females on trait X (e.g., dominance) but lower than females on trait Y (e.g., anxiety; see e.g., Kaiser et al., 2020). The line that connects the two centroids is also the axis of maximal sexual dimorphism (see Mitteroecker et al., 2015); for descriptive clarity, I label it the *centroid axis*.^1^

As shown in the figure, the sex-directionality of individual profiles is determined by their orthogonal projection on the centroid axis. The *directional boundary* is orthogonal to the centroid axis, passes through the unweighted centroid mean (i.e., the midpoint between the male and female centroids), and identifies points that lie at the same Euclidean distance from the male and female centroids; the corresponding profiles are neither male- nor female-directional and have an M-F_D_ score of zero. Male-directional profiles lie on the masculine side of the boundary (i.e., they are closer to *M* than to *F* according to the Euclidean distance); by convention, they correspond to positive values of M-F_D_. Female-directional profiles lie on the feminine side and are indicated by negative M-F_D_ values. In Fig. 1, profiles *a*, *b*, and *c* are all male-directional; *a* is more male-directional than *b* and *b* is more male-directional than *c*. Profiles *e* and *d* are female-directional, and have the same sex-directionality (i.e., the same projection on the centroid axis). Finally, profile *f* lies on the directional boundary and is neither male- nor female-directional.

Following this definition of sex-directionality, an individual’s M-F_D_ score is simply a linear combination of his/her trait scores, centered at the unweighted mean of the two sexes (so that M-F_D_ = 0 at the directional boundary) and weighted by the mean sex difference on each trait.^2^ When the variables in the set are measured in heterogeneous and/or arbitrary units (as with most psychological traits), it is usually advisable to convert them to standardized scores, yielding:

1 $${{\text{M-F}}}_{{\text{D}}}={\mathbf{z}}^{{\text{T}}}\mathbf{d}\frac{1}{\sqrt{{\mathbf{d}}^{{\text{T}}}\mathbf{d}}}={\mathbf{z}}^{{\text{T}}}\mathbf{d}\frac{1}{\Vert \mathbf{d}\Vert },$$

where** z** is a column vector of trait scores, standardized by the pooled within-sex *SD* of each trait and centered on the unweighted mean of the male and female distributions; and** d** is a vector of Cohen’s *d* values for the same traits (Cohen’s *d* is the mean sex difference standardized by the pooled within-sex *SD*; positive values indicate higher means in males). Note that the norm $\Vert \mathbf{d}\Vert$ corresponds to the standardized Euclidean distance between the male and female centroids. Normalizing by $\frac{1}{\Vert \mathbf{d}\Vert }$ yields sex-directionality scores that are scaled in a way analogous to sex-typicality scores (M-F_T_) obtained by linear discriminant analysis (see below). The R function *mf.indices* that accompanies this paper uses Eq. 1 to calculate M-F_D_ scores.

### Sex-typicality

From a different and complementary perspective, masculinity-femininity can be construed as a measure of relative typicality with respect to the male versus female distributions. A person is more masculine than another to the extent that his/her trait profile is more characteristic of males and less characteristic of females (vice versa for femininity). Even if men have broader shoulders than women on average, a person with narrower shoulders will be rated as more physically masculine than one with broader shoulders provided that the width of his/her shoulders combines with other traits (such as height) into a kind of profile that is relatively more typical of males than of females. In comparison with sex-directionality, the focus shifts from trait combinations that maximize the size of sex differences to combinations that maximize the *statistical separation* between the sexes—and, consequently, the ability to correctly classify an individual as male or female based on his/her trait profile. In line with previous contributions (see Del Giudice, 2022), I use *sex-typicality* (M-F_T_) for indices that map individual profiles on a continuum of maximal separation, analogous to the continuum of maximal dimorphism that underlies sex-directionality. When profile typicality is used to estimate the probability that a person is male or female—as in gender diagnosticity—I propose the more specific label of *sex-probability* (M-F_P_; more on this below).

**Fig. 2:**
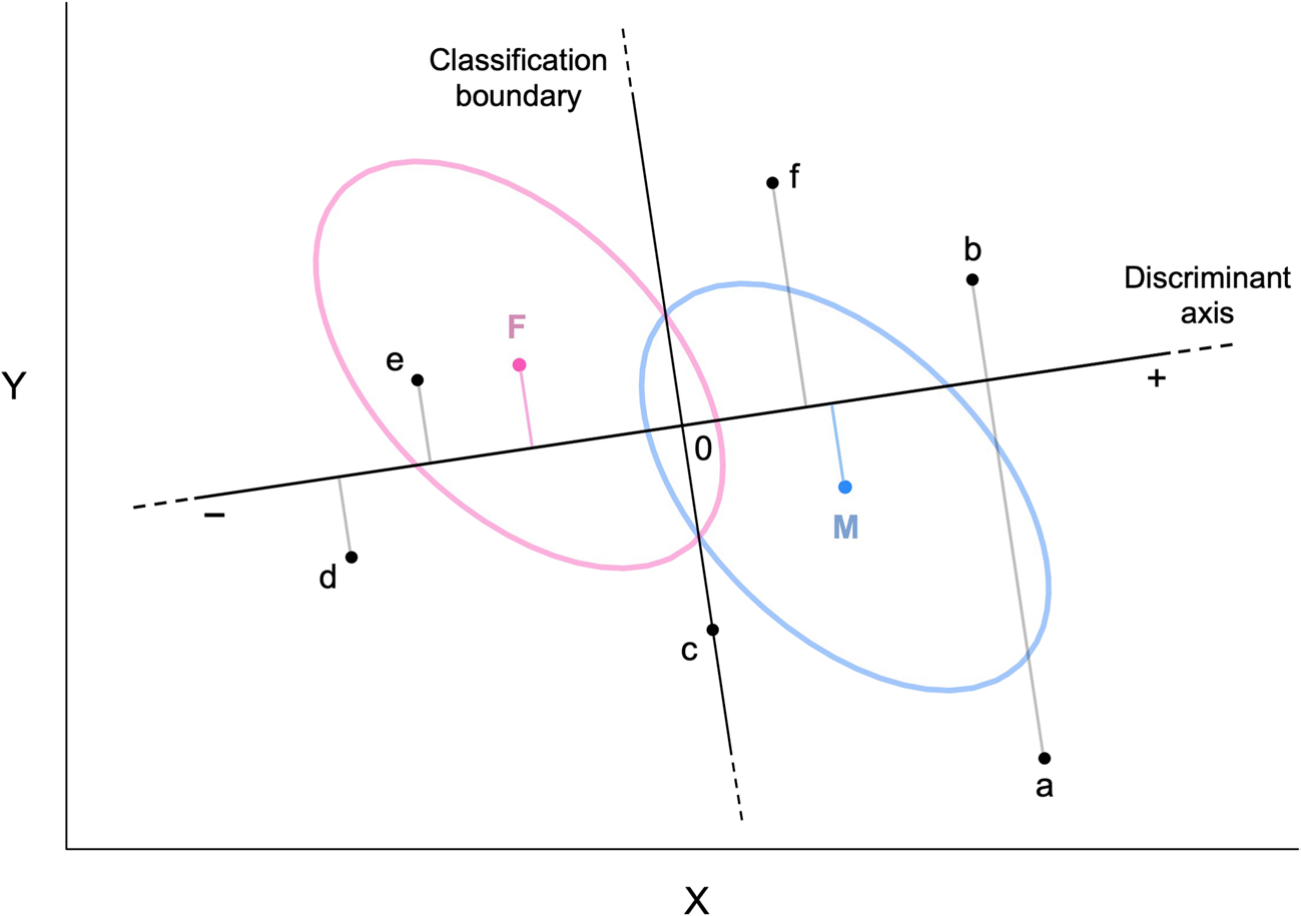
Schematic illustration of sex-typicality (based on LDA) with two correlated traits X and Y. The distributions and points shown in the figure are the same as in Fig. 1

The default approach for computing M-F_T_ scores is to make the simplifying assumption that the data are multivariate normal, with equal covariance matrices in the two sexes (e.g., Verweij et al., 2016; see Del Giudice, 2022). In this scenario, the axis that maximizes the statistical separation between the sexes (or, equivalently, minimizes their overlap) is not the centroid axis but the *discriminant axis*. The sex-typicality of individual profiles is determined by their orthogonal projection on the discriminant axis, as illustrated in Fig. 2. The *classification boundary* is orthogonal to the discriminant axis and identifies points that lie at the same Mahalanobis distance^3^ from the male and female centroids (M-F_T_ = 0); assuming equal proportions of males and females in the population, both the classification boundary and the directional boundary pass through the unweighted centroid mean. Male-typical profiles (positive M-F_T_ scores) are closer to *M* than to *F* according to the Mahalanobis distance, and are more likely to be males than females; female-typical profiles (negative M-F_T_ scores) are closer to *F* and more likely to be females than males.^4^

Figure 2 shows the same profiles of Fig. 1, along with their projections on the discriminant axis. Profiles *a*, *b*, and *f* are male-typical; *a* and *b* have the same sex-typicality and are both more male-typical than *f*. Profiles *d* and *e* are female-typical, whereas profile *c* lies on the classification boundary and is neither male- nor female-typical.

Based on the assumptions laid out in the preceding paragraph, the linear combination that yields M-F_T_ scores corresponds to the *discriminant function* of linear discriminant analysis (LDA; see Boedeker & Kearns, 2019; Venables & Ripley, 2002). Trait scores are centered at the unweighted mean of the two sexes and weighted by a vector of discriminant coefficients **a**. For standardized scores, the discriminant coefficients are obtained as:

2 $$\mathbf{a}={\mathbf{R}}^{-1}\mathbf{d},$$

where **R** is the pooled within-sex correlation matrix. It is convenient to normalize the discriminant scores by $\frac{1}{\sqrt{{\mathbf{a}}^{{\text{T}}}{\mathbf{R}}^{-1}\mathbf{a}}}$ so that their within-sex variance equals 1 (Venables & Ripley, 2002). This yields:

3 $${{\text{M-F}}}_{{\text{T}}}={\mathbf{z}}^{{\text{T}}}\mathbf{a}\frac{1}{\sqrt{{\mathbf{a}}^{{\text{T}}}{\mathbf{R}}^{-1}\mathbf{a}}}.$$

If traits are all orthogonal (**R** = **R**^–1^ =** I**), the Mahalanobis distance reduces to the standardized Euclidean distance (see Del Giudice, 2023a); as a result, **a** = **d** and the M-F_T_ scores computed with Eq. 3 become identical to the M-F_D_ scores computed with Eq. 1.

It is important to stress that LDA is not the only method that may be used to calculate M-F_T_ scores. A natural alternative to consider is logistic regression, which is structurally equivalent to LDA but does not assume multivariate normality (see James et al., 2021). By default, function *mf.indices* calculates M-F_T_ scores with LDA (Eq. 3), with the option of using logistic regression instead.^5^ In principle, one could also compute M-F_T_ indices based on nonlinear discriminant analysis (e.g., Roth & Steinhage, 1999), or other methods that maximize the separation between the sexes in a nonlinear transformation of the original trait space; to the best of my knowledge, these nonlinear methods have yet to be applied to the analysis of masculinity-femininity.

#### Sex-typicality versus sex-directionality: Rank reversals and discordant profiles

While sex-typicality and sex-directionality are both meaningful aspects of masculinity-femininity, they do not measure exactly the same thing. In most realistic scenarios, the M-F_T_ and M-F_D_ scores of Eqs. 1 and 3 are going to be strongly and positively correlated (i.e., the angle between the centroid and discriminant axes is going to be much less than 90º), but that correlation is *not* going to be perfect except in special cases (for example, when the traits under study are all orthogonal). This has some interesting implications for the classification and ranking of individual profiles, as I now discuss.

**Fig. 3:**
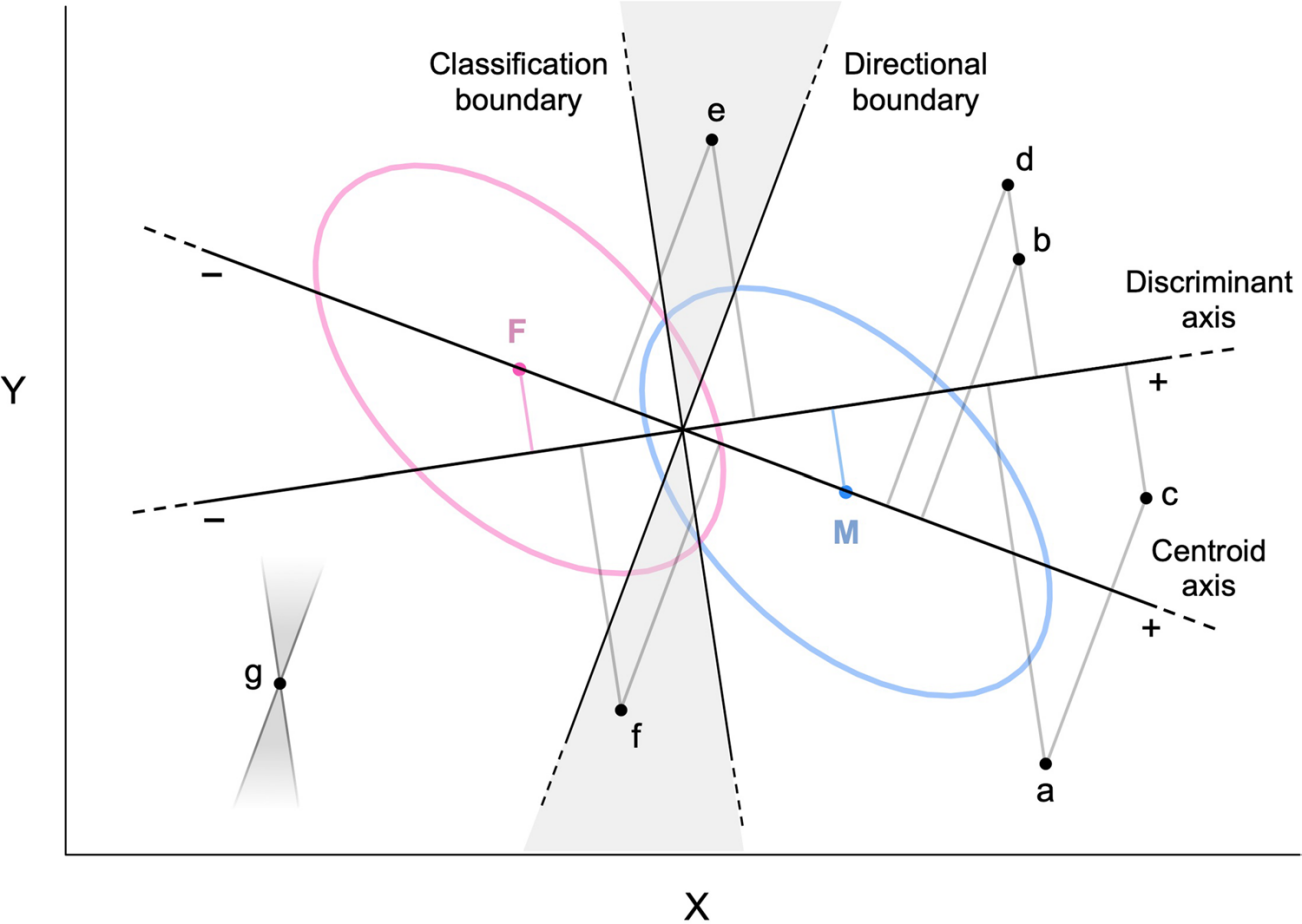
Schematic illustration of the relations between sex-directionality and sex-typicality (based on LDA) with two correlated traits X and Y

Figure 3 shows the same distributions of traits X and Y of Figs. 1 and 2, but the centroid and discriminant axes (with the corresponding boundaries) are both depicted at the same time. A new set of points offers a geometric illustration of the phenomena that can take place at the interface of sex-typicality and sex-directionality. Consider profile *b*, which is *less* male-directional than *a* and yet *more* male-typical. If X and Y represent dominance and anxiety (respectively), *a* is somewhat more dominant than *b* and considerably less anxious; both differences go in a more masculine direction when examined separately. However, the *combination* of *a*’s dominance and anxiety scores is less male-typical than the combination of *b*’s scores—specifically, *a* is much less dominant than one would expect given its low level of anxiety. For comparison, profile *c* is just as male-directional as *a,* but more male-typical than both *a* and *b*; whereas profile *d* is as male-typical as *a,* but less male-directional than both *a* and *b*.

The example of *a* and *b* demonstrates how the ranking of two profiles can reverse depending on whether one considers sex-typicality or sex-directionality. An even more dramatic pattern of inconsistency is illustrated by profile *e*, which is simultaneously *female*-directional and *male*-typical. Conversely, profile *f* is male-directional but female-typical. In fact, any profile lying in the shaded regions between the directional and classification boundaries will show a discordance between sex-typicality and sex-directionality. And the wider the angle between the discriminant and centroid axes (i.e., the smaller the correlation between M-F_T_ and M-F_D_ scores), the larger the proportion of profiles that can be expected to exhibit discordant M-F patterns. Discordant profiles are characterized by M-F_T_ and M-F_D_ scores in the vicinity of zero, neither strongly masculine nor strongly feminine. Profiles that fall in this category are interesting because they may serve as “test cases” to probe the relative influence of sex-typicality and sex-directionality (for example, on perceptions of masculinity-femininity in a certain domain). In practice, however, one has to consider that discordant M-F patterns are easily overshadowed by even small amounts of measurement error, and must be treated with caution unless traits have been measured with very high levels of reliability.

Of note, the “local” geometry of rank reversals reproduces the “global” geometry of discordant profiles: each point in the multivariate space can be seen as lying at the intersection of two boundaries with the same orientation as the directional and classification boundaries; those boundaries separate the points that rank consistently with the focal point from those that exhibit rank reversal. Figure 3 illustrates this concept in the case of profile *g*. All the profiles that lie in the shaded regions originating from *g* (shown only in part) will switch rank with *g* depending on whether M-F_T_ and M-F_D_ scores are considered; whereas the profiles that populate the rest of the space will show consistent rankings with *g* regardless of the chosen index.

### Sex-probability

The indices of sex-typicality discussed in the previous section are useful because they locate profiles on a continuum that is analogous to that of sex-directionality. Among other things, this facilitates geometrical comparisons between different aspects of masculinity-femininity (as in Fig. 3) and thus promotes conceptual clarity. However, in some cases it can be convenient to translate the dimensional concept of typicality into a more intuitive notion: the probability of being classified as male (vs. female) based on one’s combination of traits.

Gender diagnosticity (Lippa, 1991, 1998, 2001; Lippa & Connelly, 1990) was the first method to employ classification probabilities to measure masculinity-femininity; GD scores are the prototypical example of a sex-probability index (M-F_P_). In the original implementation of GD, probabilities are estimated with LDA. Assuming equal prior probabilities of being male versus female (see Lippa, 1991), the relevant formula is:

4 $${{\text{M-F}}}_{{\text{P}}}=\frac{{\text{exp}}({\mathbf{z}}^{{\text{T}}}\mathbf{a})}{1+\mathrm{ exp}({\mathbf{z}}^{{\text{T}}}\mathbf{a})}.$$

Note that, in Eq. 4, probabilities are calculated from the entire set of traits at once. In the GD literature, researchers often calculate multiple probability estimates from subsets of traits (e.g., preferences for different sets of occupations) and average them, as a means to estimate the reliability of the resulting scores (e.g., Lippa, 1991; Lippa & Connelly, 1990).

**Fig. 4:**
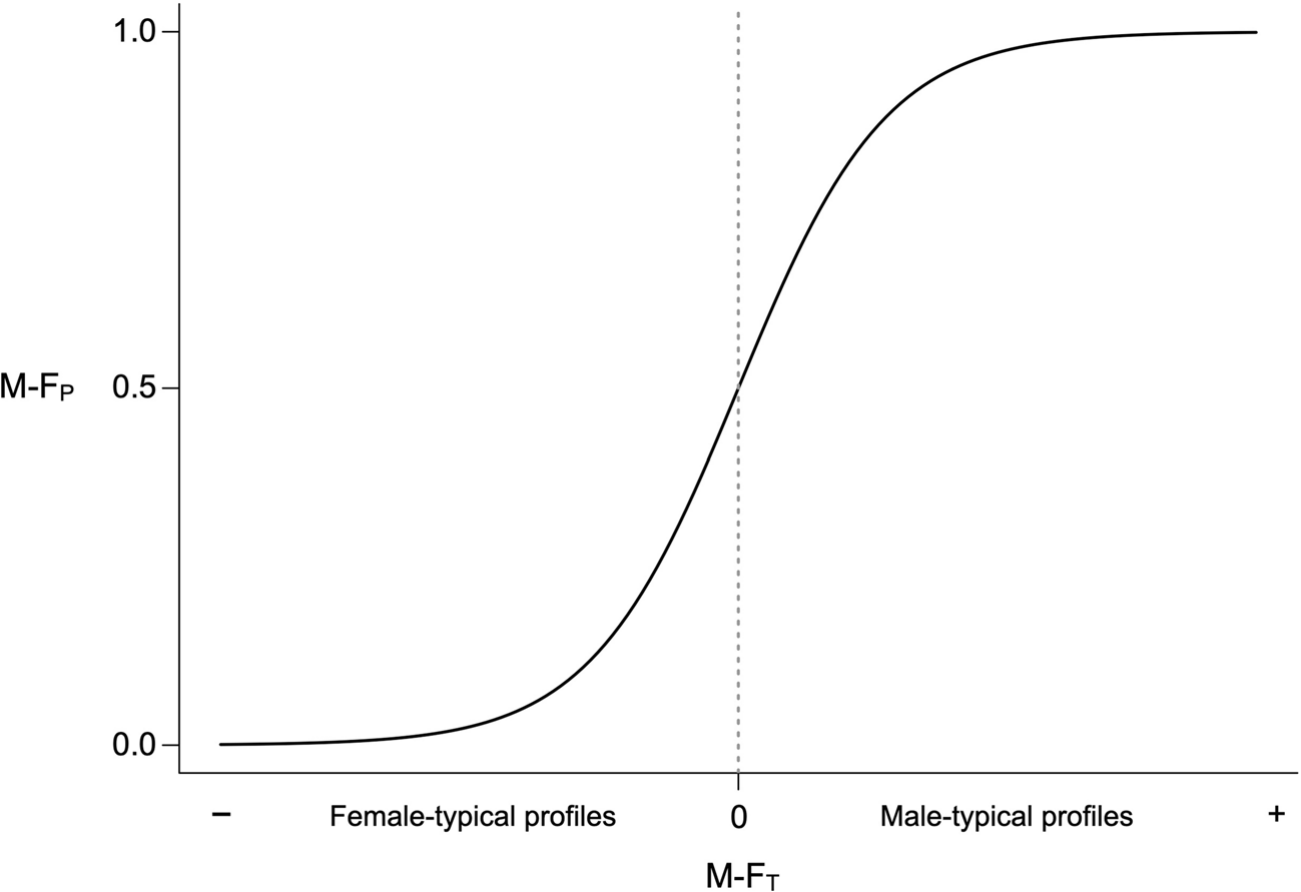
In both LDA and logistic regression, sex-probability (M-F_P_) is linked to sex-typicality (M-F_T_) by a logistic function. Negative (female-typical) M-F_T_ scores correspond to M-F_P_ < 0.5, whereas positive (male-typical) scores correspond to M-F_P_ > 0.5

Since LDA models distributions as multivariate normal, one may wish to relax this assumption and use logistic regression instead, as was done by Ilmarinen et al. (2023). This amounts to replacing the un-normalized discriminant score ${\mathbf{z}}^{{\text{T}}}\mathbf{a}$ in Eq. 4 with the un-normalized linear predictor of the regression model. Function *mf.indices* employs Eq. 4 by default, with the option of using logistic regression as an alternative. In both cases, M-F_P_ scores are a simple monotonic (logistic) function of the corresponding M-F_T_ scores, as visualized in Fig. 4. With equal priors for males and females, a sex-typicality score of zero (indicating that the profile lies on the classification boundary) corresponds to a sex-probability of 0.5. Male-typical profiles are also male-probable (M-F_P_ > 0.5), whereas female-typical profiles are also female-probable (M-F_P_ < 0.5). This means that the direction of sex-probability is always concordant with that of sex-typicality (there can be no rank reversals or discordant profiles); also, the patterns of reversal and discordance that take place between sex-typicality and sex-directionality are exactly mirrored in the comparison between sex-probability and sex-directionality.

With the proliferation and widespread adoption of machine learning methods, the options for calculating other variants of M-F_P_ indices—based on models that can range from simple to extremely complex—have greatly expanded. For example, Sanchis-Segura et al. (2022) used brain morphology data to compute what they called the “probability of being classified as male” (PCAM) with five classifiers: LDA, logistic regression, multiple adaptive regression splines (MARS), support vector machines (SVM), and random forests (for an overview of these methods see James et al., 2021). The sex-probability scores generated by the five classifiers were strongly correlated (*r*s from .79 to .99) when sex differences in total brain volume were not controlled for, and moderately to strongly correlated (*r*s from .56 to .99) after the relevant correction. Unlike LDA or logistic regression, some of these methods yield an M-F_P_ score *without* a corresponding M-F_T_ score. For example, random forests are ensemble models composed of a large number of simpler classification trees (see James et al., 2021); the standard way of obtaining a sex-probability score from a random forest is to “count the votes” of individual trees, and use the proportion of “male” classifications over the entire ensemble as a measure of probability.^6^

In sum, the main advantages of sex-probability indices are their interpretability and the fact that they can be easily obtained from a wide range of classification models. A potential downside is that, compared with male-typicality indices, they tend to compress the masculine and feminine ends of the continuum into a narrow range of values (see Fig. 4). This is a virtue when the task is binary classification, but not necessarily when one seeks to measure individual differences on a common scale. In particular when the male and female distributions are statistically well separated, M-F_P_ scores provide good discriminability for intermediate scores close to 0.5, but tend to blur the distinction between profiles that are “merely” male- or female-typical and those that are highly or extremely typical of one sex. Depending on the application at hand, this may or may not be an issue. For example, as I discuss later, sex-probability scores can be less sensitive to measurement error than their counterparts under certain scenarios. It is also possible that, in some research contexts, variation at the tails of the typicality distribution is less meaningful and/or predictive than variation around the classification boundary, making sex-probability indices the preferred option. In any event, one should keep in mind that M-F_P_ and M-F_T_ scores have different statistical properties and potentially different costs and benefits.

### Sex-centrality

From the perspective of sex-typicality, a trait profile is deemed more or less characteristic of males/females based on its position on the axis of maximal statistical separation between the sexes. However, there is an alternative way to think about representativeness that leads to yet another type of M-F index. If one takes the male and female centroids (i.e., the average male and female profiles) as being maximally representative of their respective sexes, a profile can be rated as masculine/feminine by comparing its distance from the male centroid with its distance from the female centroid. This is the notion of masculinity-femininity as *sex-centrality* (M-F_C_). A profile is male-central to the extent that it is more “average” relative to the male distribution than to the female distribution (and vice versa for female-central profiles). Thus, sex-centrality can be useful to distinguish the relative averageness of a profile from its extremity or typicality (attractiveness comes to mind as a potential area of application, given that faces with average features tend to be rated as attractive).

Under multivariate normality, the Mahalanobis distance (see Footnote 3) provides a natural way to measure multivariate distances when traits are correlated. This can be leveraged to derive a simple index of sex-centrality. Taking the difference between the distance from *F* and that from *M* yields a positive score when a profile is closer to the male centroid (male-central), negative when a profile is closer to the female centroid (female-central). To make scores more interpretable, this difference can be normalized by its maximum possible value, which corresponds to the Mahalanobis distance between the centroids. This yields:

5 $${{\text{M-F}}}_{{\text{C}}}=\frac{{D}_{{\text{M}}}\left(\mathbf{z}, \mathbf{f}\right)-{D}_{{\text{M}}}\left(\mathbf{z}, \mathbf{m}\right)}{{D}_{{\text{M}}}\left(\mathbf{m}, \mathbf{f}\right)},$$

where *D*_M_ is the Mahalanobis distance between two points (based on the pooled correlation matrix), and **m** and **f** are the centered and standardized trait vectors corresponding to the male and female centroids. Function *mf.indices* uses Eq. 5 to calculate M-F_C_ scores. A score of M-F_C_ = 1 means that a profile is as male-central as the male centroid *M*; a score of M-F_C_ = –1 means that a profile is as female-central as the female centroid *F*. Profiles that lie at the same Mahalanobis distance from the two centroids have M-F_C_ = 0. Note that the line of points with M-F_C_ = 0 is nothing but the familiar classification boundary; male-central profiles (M-F_C_ > 0) are also male-typical and male-probable, whereas female-central profiles (M-F_C_ < 0) are also female-typical and female-probable. In other words, the direction of sex-centrality is always concordant with that of sex-typicality and sex-probability if these constructs are based on LDA. (As noted earlier, logistic regression typically yields very similar results despite its different assumptions.)

**Fig. 5:**
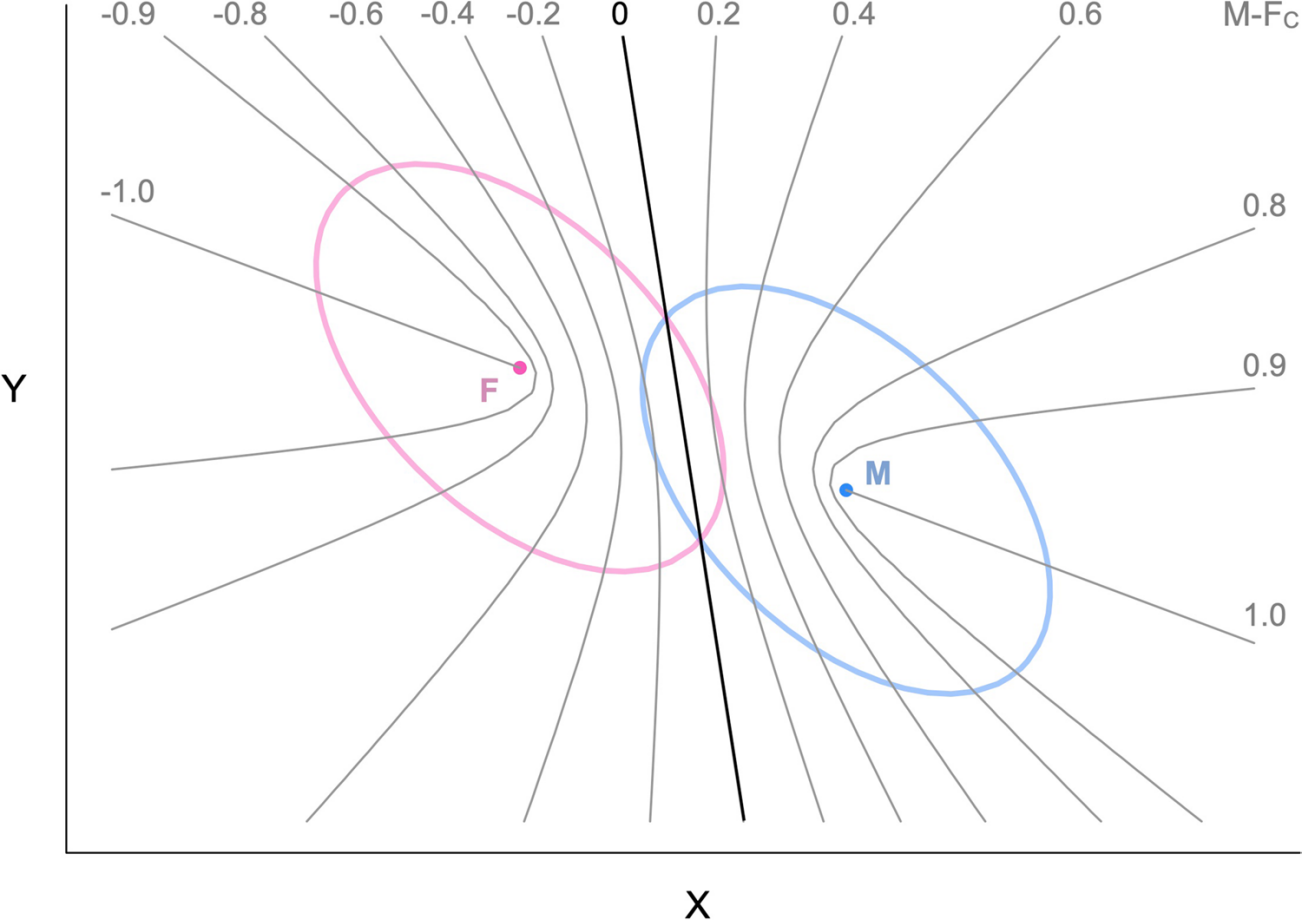
Schematic illustration of sex-centrality (M-F_C_) with two correlated traits X and Y. The gray curves connect points with the same value of M-F_C_. *Note:* the specific values of M-F_C_ shown in the figure are based on distributions with a correlation of –.50 between X and Y, and a Mahalanobis distance of 1.62 between the centroids

In contrast with the other M-F indices described so far, the sex-centrality of a profile cannot be described by a simple orthogonal projection on a particular axis. Figure 5 illustrates this point by showing a selection of curves connecting points with the same value of M-F_C_ (or “iso-centrality” curves). As one moves away from the classification boundary, the curvature progressively increases, until the curves collapse into two half-lines (corresponding to M-F_C_ = ±1) originating from the centroids and aligned with the directional axis. The key properties of M-F_C_ can be gleaned from Fig. 5. For intermediate scores around zero, iso-centrality curves remain roughly parallel to the classification boundary; hence, in this region, there are going to be strong correlations between M-F_C_, M-F_T_, and M-F_P_. (Recall that this is the same region in which M-F_P_ scores are approximately linearly proportional to M-F_T_; see Fig. 4). However, the behavior of M-FC diverges more and more dramatically from that of M-F_T_ and M-F_P_ as one moves toward the masculine and feminine ends of these indices.

One consequence is that, even if the direction of sex-centrality is always concordant with that of sex-typicality, there is room for rank reversals (e.g., a profile can be more female-typical but less female-central than another). Another is that sex-centrality scores are less “compressed” toward the extremes than their sex-probability counterparts. Across the full distribution of scores, M-F_C_ tends to correlate strongly with M-F_T_, even when the male and female distributions are highly separated (some examples below). This makes it largely redundant in many research contexts. However, when researchers are specifically interested in the extremes of masculinity-femininity, M-F_C_ provides unique information and can usefully complement other, more standard indices such as M-F_T_ and M-F_D_.

#### Sex-centrality in high-dimensional domains

As I noted at the start of this section, the meaning of sex-centrality rests on the notion that centroids are maximally representative of their distributions. This is true in the sense that, as multivariate means, they are the points with the highest probability density (at least in normal and other bell-shaped distributions). In low-dimensional contexts, it is also the case that the mass of the distribution clusters around the centroid, with only a small proportion of points located in the tails. But as dimensionality increases, a larger proportion of the probability mass becomes concentrated in the *tail* region, where density is comparatively low. That is, the majority of the points move far away from the centroid, along a progressively thinner “shell” that envelopes a mostly empty interior (see Del Giudice, 2023a; Giraud, 2015; van Tilburg, 2019). As the number of traits grows larger, the male and female centroids become less representative of the majority of males and females, and even highly “sex-central” profiles are likely to lie at a considerable distance from the nearest centroid. This caveat should be kept in mind when interpreting M-F_C_ scores calculated from high-dimensional data.

## The impact of measurement error and possible remedies

In real-world datasets, traits are always measured with a smaller or greater amount of noise. The effects of measurement error on statistical M-F indices are surprisingly far-reaching, so they have to be addressed explicitly and discussed in some detail. For the present purposes, I define the *reliability* of a trait as the proportion of that trait’s observed variance that is not accounted for by measurement error (“true score variance” in classical test theory). I also define the *validity* of an M-F index as the correlation between its measured values and the “true” values that it would obtain if the traits had been measured without error (that is, with perfect reliability).

To begin, one should note that measurement error attenuates the correlations among traits, shrinking them toward zero (see Del Giudice, 2022). This is important because, as the reliability of the variables decreases, the observed correlation matrix becomes more similar to the identity matrix, and the standardized discriminant axis moves closer to the centroid axis. The result is that indices of sex-typicality, sex-probability, and sex-centrality all become less clearly distinct from sex-directionality; M-F_T_, M-F_P_, and M-F_C_ scores become more highly correlated (and thus more redundant) with M-F_D_ scores, and the proportion of discordant profiles (those in shaded regions of Fig. 3) diminishes accordingly. This phenomenon is not due to sampling error but to measurement error, and therefore is *not* ameliorated by increasing the size of the sample.

A notable implication is that measurement error reduces the validity of sex-typicality and sex-centrality indices (both of which rely on patterns of trait correlations) much more dramatically than that of sex-directionality. Sex-probability as defined in Eq. 4 is just a monotonic function of sex-typicality; however, high and low typicality values are compressed when turned into probabilities, especially when there is little overlap between the male and female distributions. As a result, M-F_P_ scores may show higher or lower validity than M-F_T_ and M-F_C_ scores, depending on the specific patterns found in the data. Whenever alternative M-F indices are directly compared in a statistical analysis (for example, to assess their relative predictive value with respect to an outcome), one should keep in mind that their validities are going to differ in predictable ways, especially if traits have been measured with substantial noise. The problem of validity is going to become especially acute if M-F indices are calculated from collections of single items, which tend to have much lower reliabilities than longer psychometric scales.

**Fig. 6:**
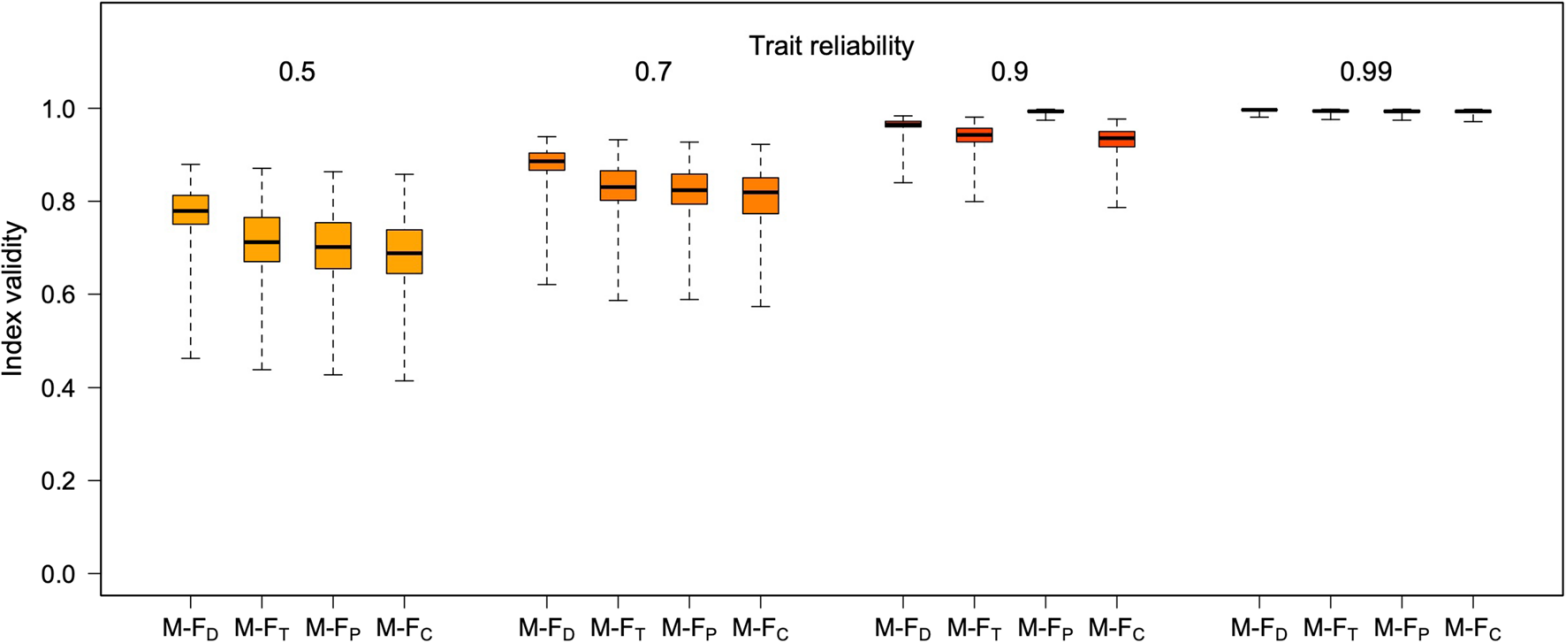
Validity of the four M-F indices at different levels of trait reliability, with five traits. Boxplots summarize the distribution of results across 100 simulated samples (*N* = 2000 each, 50% females). The mean absolute true correlations between traits were in the .20–.25 range; univariate sex differences (Cohen’s *d*) were normally distributed, with mean 0 and SD = 0.70, and the true Mahalanobis distance between the male and female centroids had an average of about 2

**Fig. 7:**
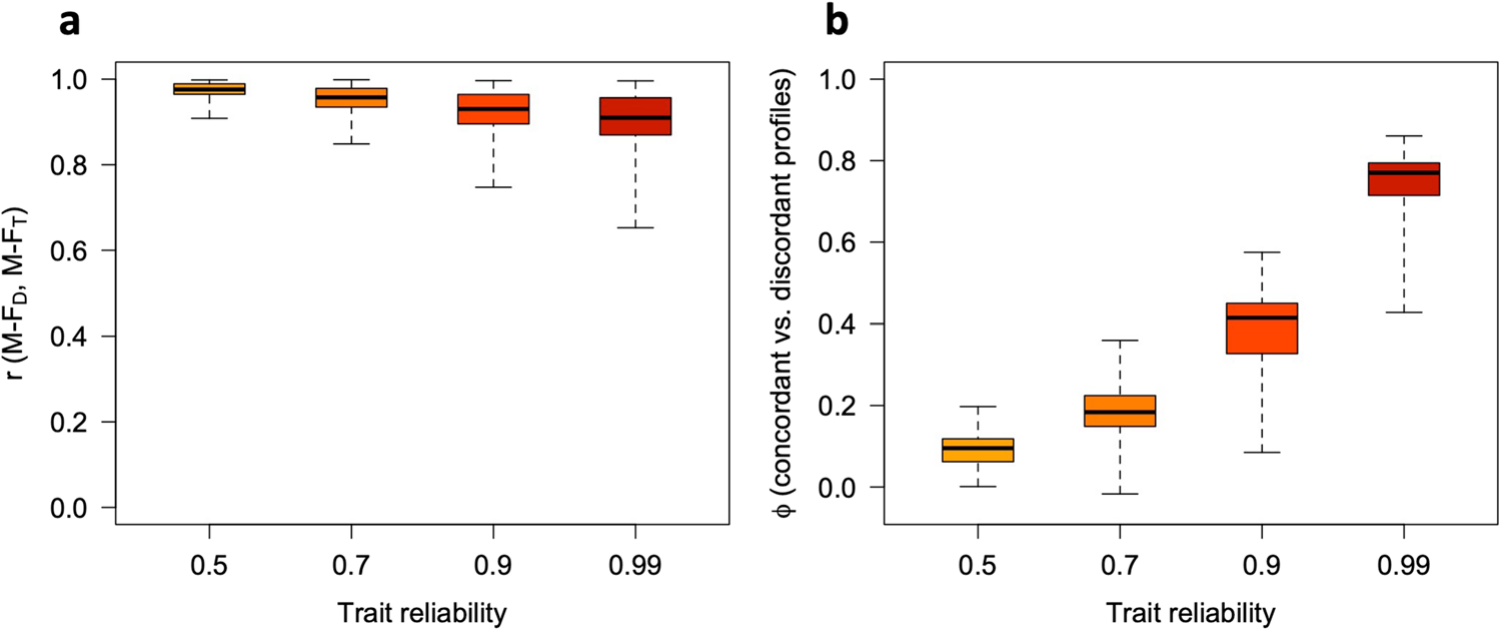
Relations between sex-directionality (M-F_D_) and sex-typicality (M-F_T_) at different levels of trait reliability, with five traits. Panel (**a**): observed correlations between M-F_D_ and M-F_T_. Panel (**b**): Phi coefficients for concordant vs. discordant profiles (i.e., profiles showing M-F_D_ and M-F_T_ scores with the same or opposite signs) at different levels of trait reliability. All simulation parameters were the same as in Fig. 6

**Fig. 8:**
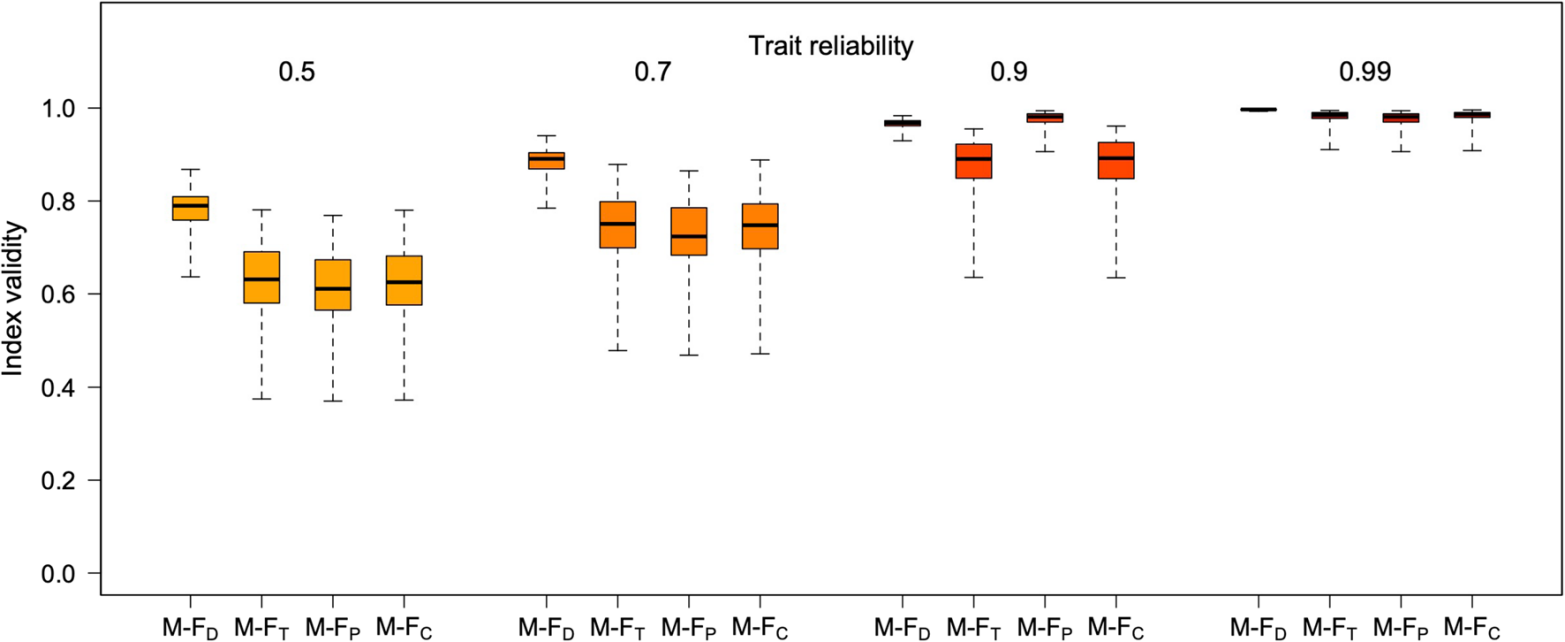
Validity of the four M-F indices at different levels of trait reliability, with 10 traits. Boxplots summarize the distribution of results across 100 simulated samples (*N* = 2000 each, 50% females). The mean absolute true correlations between traits were in the .20–.25 range; univariate sex differences (Cohen’s *d*) were normally distributed, with mean 0 and SD = 0.50, and the true Mahalanobis distance between the male and female centroids had an average of about 3

**Fig. 9:**
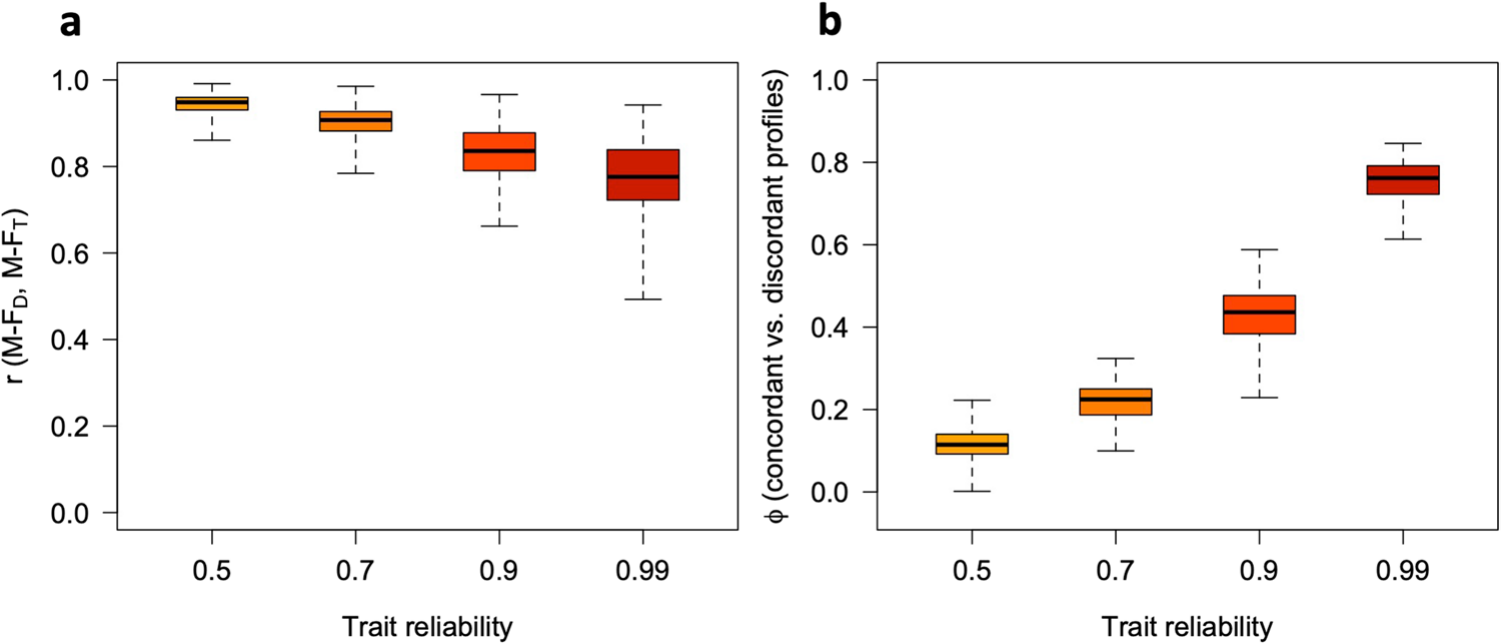
Relations between sex-directionality (M-F_D_) and sex-typicality (M-F_T_) at different levels of trait reliability, with 10 traits. Panel (**a**): observed correlations between M-F_D_ and M-F_T_. Panel (**b**): Phi coefficients for concordant vs. discordant profiles (i.e., profiles showing M-F_D_ and M-F_T_ scores with the same or opposite signs) at different levels of trait reliability. All simulation parameters were the same as in Fig. 8

**Fig. 10:**
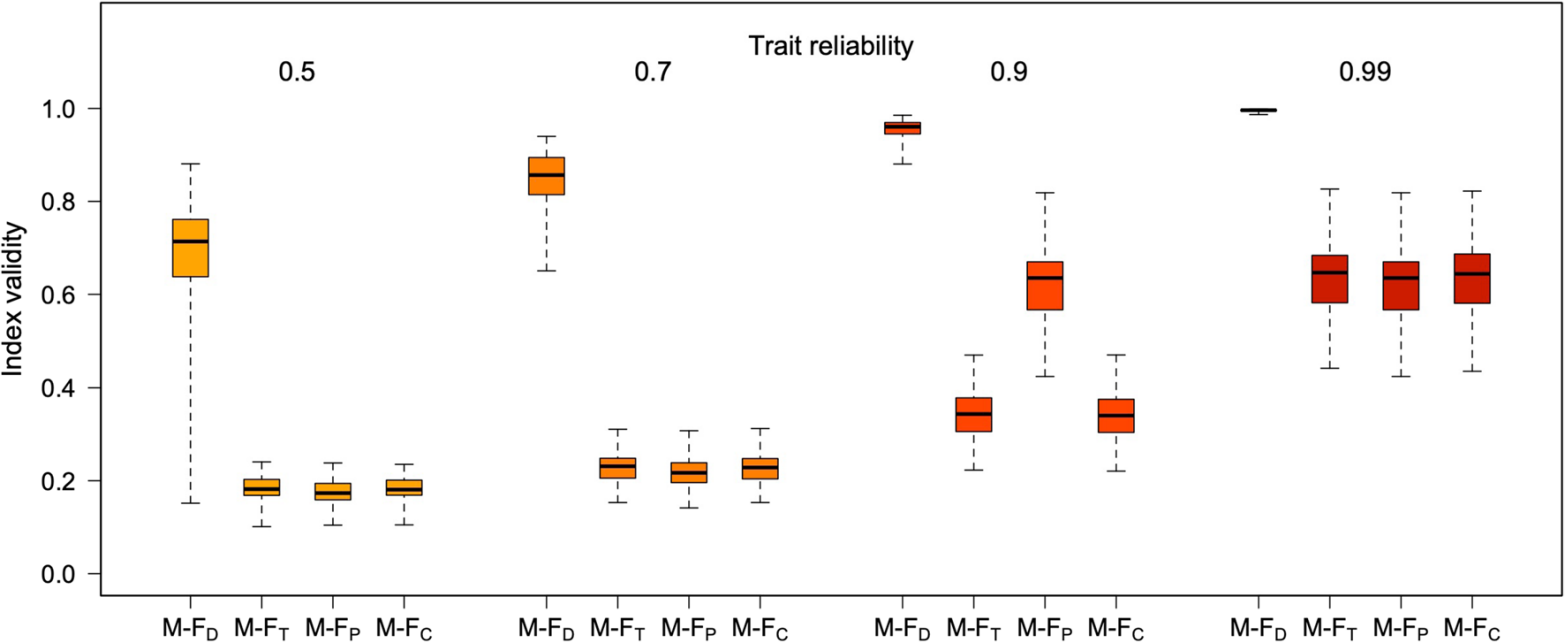
Validity of the four M-F indices at different levels of trait reliability, with 30 traits. Boxplots summarize the distribution of results across 100 simulated samples (*N* = 2000 each, 50% females). The mean absolute true correlations between traits were in the .20–.25 range; univariate sex differences (Cohen’s *d*) were normally distributed, with mean 0 and SD = 0.05, and the true Mahalanobis distance between the male and female centroids had an average of about 4

**Fig. 11:**
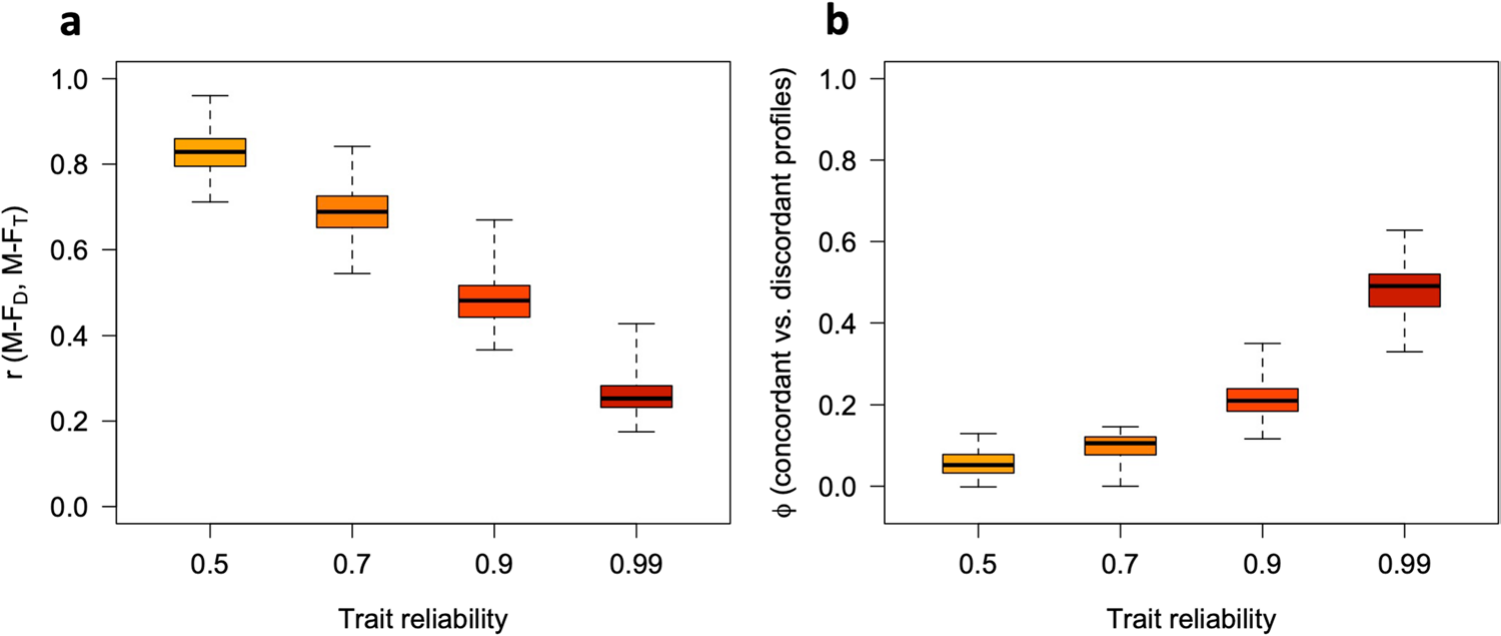
Relations between sex-directionality (M-F_D_) and sex-typicality (M-F_T_) at different levels of trait reliability, with 30 traits. Panel (**a**): observed correlations between M-F_D_ and M-F_T_. Panel (**b**): Phi coefficients for concordant vs. discordant profiles (i.e., profiles showing M-F_D_ and M-F_T_ scores with the same or opposite signs) at different levels of trait reliability. All simulation parameters were the same as in Fig. 10

Figures 6, 7, 8, 9, 10, and 11 illustrate these patterns using simulated, multivariate normal datasets with different numbers of traits (from 5 to 30) and levels of trait reliability (from .50 to .99). Correlation matrices were generated with the vine method, keeping the beta parameter fixed at 4 (see Lewandowski et al., 2009). To reflect typical real-world scenarios, the true Mahalanobis distance between the male and female centroids was allowed to increase with the number of traits in the dataset (as detailed in the figure legends). All the M-F indices were calculated with LDA using function *mf.indices*.

As can be seen in Figs. 6, 7, 8, 9, 10, and 11, increasing the number of traits amplifies the adverse impact of noise—particularly on M-F_T_, M-F_C_, and (somewhat less consistently) M-F_P_. This suggests one possible response to measurement error, which is to minimize the number of traits in the analysis and/or aggregate them into a smaller number of composites with higher reliability (e.g., via factor analysis or principal component analysis [PCA]). While this approach can be quite effective, reducing the number of traits can have its own downsides; for example, if sex differences in a certain domain emerge more clearly at a finer level of analysis (as is the case with personality; see Del Giudice, 2022, 2023a), the aggregation of narrow traits into broader composites may easily end up obscuring them. In many cases, there is a trade-off between the granularity of the data (and hence their ability to accurately describe sex-differentiated patterns of traits) and their vulnerability to both sampling and measurement noise.

When feasible, the alternative approach is to apply an error correction procedure to the data before the analysis, to remove some noise from the trait measures and obtain a more accurate estimate of the correlation matrix. If reliability estimates for the observed trait values are available, the function *mf.indices* has the option of correcting the data using *data matrix disattenuation* (DMD), a novel correction method presented in Del Giudice (2023b). DMD can significantly increase the reliability of variables in multivariate datasets, while adjusting trait correlations to counteract the attenuating effect of measurement error. The correction afforded by DMD becomes more effective as the sample size and the number of variables increase. Indeed, when using error correction, it might pay off to *maximize* rather than minimize the number of traits in the analysis. In the online supplement, the simulated data of Figs. 6, 7, 8, 9, 10, and 11 are reanalyzed to demonstrate how this method can substantially improve the validity of M-F scores, and recover much more accurate correlations between sex-typicality and sex-directionality. However, correcting measurement error is not without costs—in particular, reducing the bias due to error increases the variance of parameter estimates, widening their standard errors (see Carroll et al., 2006; Del Giudice, 2023b). For this reason, error correction is especially advisable when sample size is large enough that the resulting inflation of sampling variance can be tolerated. Other methods that may be used to correct measurement error are described by Mansolf (2023) and Carroll et al. (2006).

As is apparent from Figs. 6, 7, 8, 9, 10, and 11, measurement error has a particularly strong impact on the classification of discordant profiles. As larger amounts of noise are added to measured trait values, more profiles that would be discordant based on their true M-F_D_ and M-F_T_ scores are classified as concordant, while more would-be concordant profiles are classified as discordant. This can greatly reduce the validity of the “discordant” category. Crucially, the validity of concordant/discordant classifications drops rather steeply as measurement error increases; even relatively small amounts of error can easily lead to a situation in which misclassified profiles outnumber the correctly classified ones. As illustrated in the online supplement, the validity of concordant/discordant classifications improves only marginally even after correcting for measurement error. Because discordant profiles are so sensitive to noise, one should be very cautious about analyzing and interpreting profile concordance/discordance unless the traits in question have been measured with suitably high precision. (Simulations may help determine how much error can be tolerated on a case-by-case basis.)

## Empirical examples

Before concluding, I illustrate the M-F indices presented in this paper with a selection of empirical datasets. All analyses were performed in R 4.2.2 (R Core Team, 2022). M-F indices were calculated and plotted with function *mf.indices*, using the default LDA method. The code and data (when available for sharing) can be downloaded at 10.6084/m9.figshare.22277758

## Masculinity-femininity in body morphology

For the first example, I calculated indices of physical masculinity-femininity in adults based on eight anthropometric variables from the US National Health and Nutrition Examination Survey (NHANES), accessed via the *nhanesA* package v. 0.6.5 (Endres, 2018). Specifically, the variables are height, upper leg length, calf circumference, upper arm length, arm circumference, waist circumference, triceps skinfold, and subscapular skinfold. I chose these variables because they had a relatively low proportion of missing cases and represented a reasonable assortment of measures tapping body size, adiposity, and muscularity. To control for overall body fat, all the variables involving circumferences (calf, arm, and waist) were residualized on body mass index (BMI) prior to the analysis. The complete cases between 18 and 40 years of age included 879 males and 976 females. The Mahalanobis distance between the centroids was *D*_M_ = 3.01 (bias-corrected *D*_Mu_ = 3.00),^7^ indicating a high degree of separation between the sexes.

**Fig. 12:**
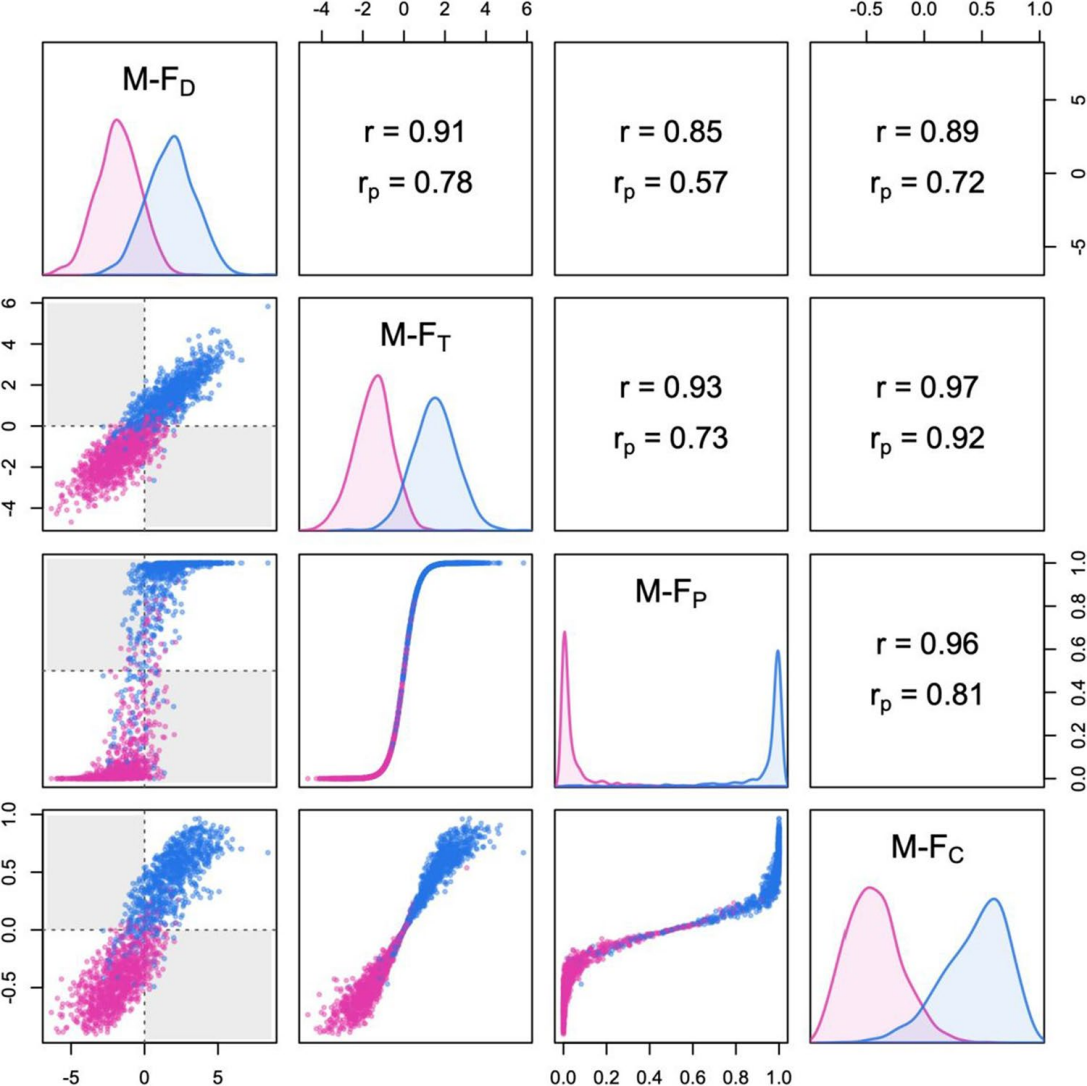
Summary plot of M-F indices measuring masculinity-femininity in body morphology (calculated with LDA based on eight variables from the NHANES dataset; see the main text for details). Correlations in the whole sample (*r*) and partial correlations controlling for sex (*r*_p_) are displayed above the diagonal. The shaded areas in the scatterplots represent discordant profiles that are male-directional but female-typical, or vice versa. M-F_D_ = sex-directionality; M-F_T_ = sex-typicality; M-F_P_ = sex-probability; M-F_C_ = sex-centrality

Figure 12 presents a graphical summary of the four M-F indices and their mutual relations. The figure shows the distribution of each index in males and females (on the diagonal), as well as bivariate scatterplots showing the individual data points, with different colors for the two sexes (below the diagonal). The shaded areas of the scatterplots in the left column identify discordant profiles, which in this case amounted to 9.6% of the sample. Above the diagonal, the figure displays the correlations among indices in the whole sample (*r*) and the corresponding partial correlations controlling for sex (*r*_p_). Partial correlations are often more meaningful and informative, as they are not confounded with the overall size of sex differences. Both sex-probability and sex-centrality tend to track sex-typicality—and one another—pretty closely; thus, the correlation between sex-typicality and sex-directionality (which also contributes to determine the proportion of discordant patterns) is of particular interest. In this dataset, the partial correlation between M-F_T_ and M-F_D_ was *r*_p_ = .78, indicating a fairly strong—but far from perfect—association between the typicality and directionality of physical profiles within each sex. As can be seen in the figure, the large separation between males and females yielded highly skewed distributions of M-F_P_ scores, narrowly clustered in the vicinity of 0 and 1.

## Masculinity-femininity in brain morphology

**Fig. 13:**
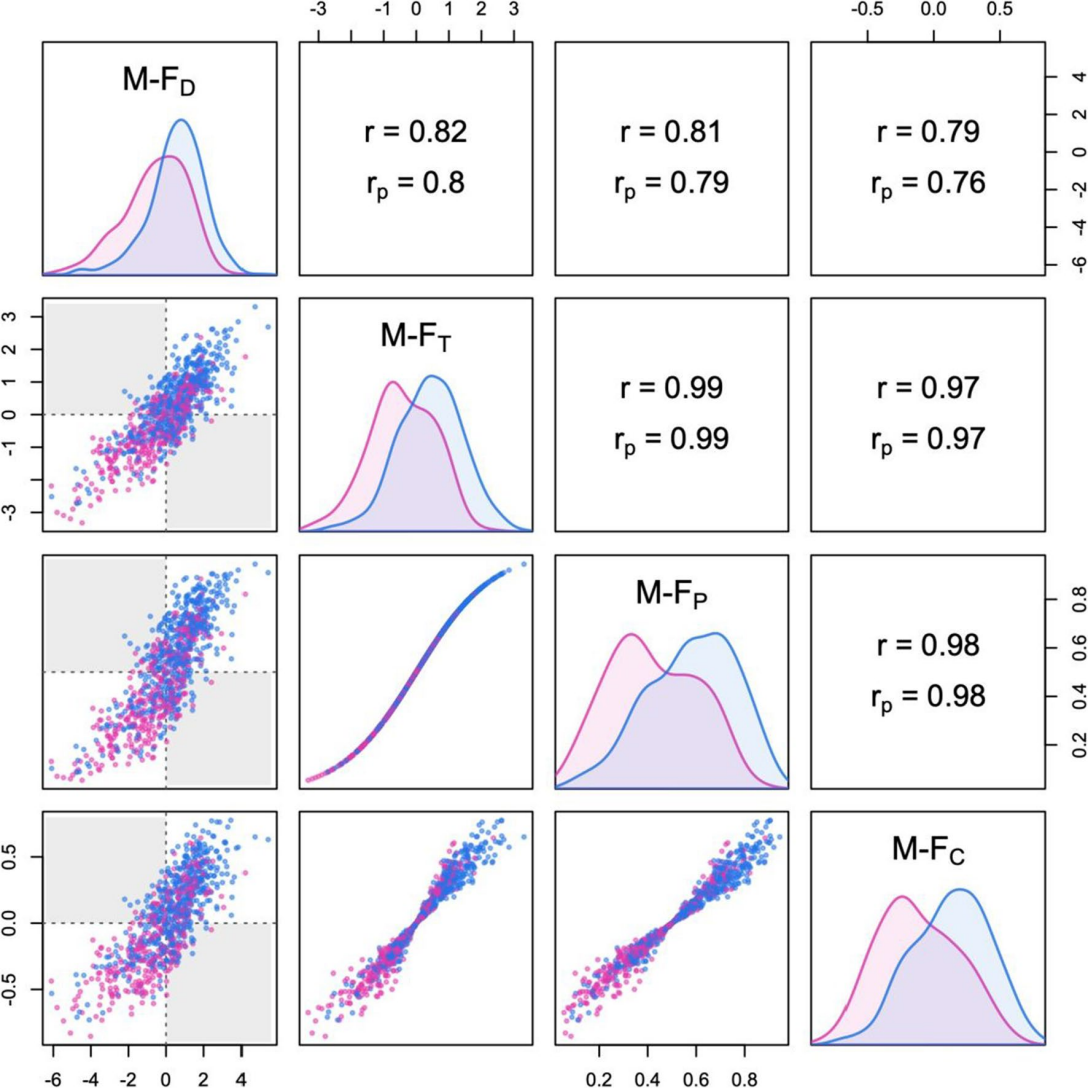
Summary plot of M-F indices measuring masculinity-femininity in brain morphology (calculated with LDA based on 11 components of gray matter volume, from the 1000 Functional Connectomes Project dataset; see the main text for details). Correlations in the whole sample (*r*) and partial correlations controlling for sex (*r*_p_) are displayed above the diagonal. The shaded areas in the scatterplots represent discordant profiles that are male-directional but female-typical, or vice versa. M-F_D_ = sex-directionality; M-F_T_ = sex-typicality; M-F_P_ = sex-probability; M-F_C_ = sex-centrality

For the second example, I used the gray matter volume data of the 1000 Functional Connectomes Project, one of the imaging datasets originally analyzed by Joel and colleagues (2015) in an influential paper on sex differences in brain morphology. The complete cases included 495 males and 360 females. In order to reduce sampling error and limit overfitting, I employed PCA followed by oblimin rotation to summarize the 116 regional variables with 11 correlated components (the number of components was suggested by parallel analysis; see Hayton et al., 2004). The results are displayed in Fig. 13. With a Mahalanobis distance of *D*_M_ = 0.86 (bias-corrected *D*_Mu_ = 0.82), the overlap between male and female brain profiles was substantially larger than in the case of physical profiles. This is clearly reflected in the distribution of M-F_P_ scores, which is markedly less skewed than in Fig. 12. Note that M-F_T_ and M-F_D_ scores were reasonably distinct, with a partial correlation of *r*_p_ = .80; accordingly, 21.4% of the brains in the dataset showed discordant M-F profiles.

## Masculinity-femininity in personality traits

**Fig. 14:**
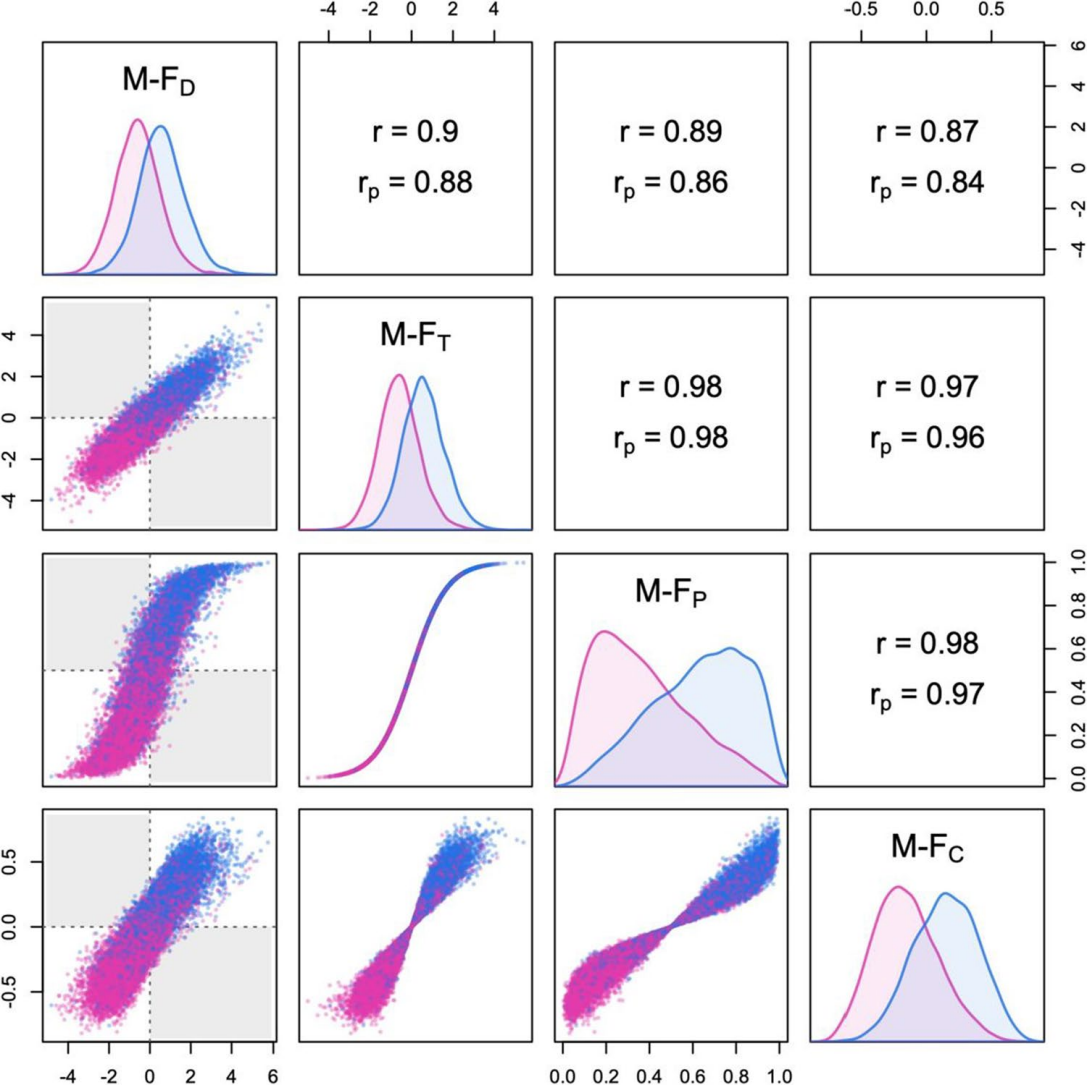
Summary plot of M-F indices measuring masculinity-femininity in personality traits (calculated with LDA based on 15 personality factors of Cattell’s 16PF model, from the Open Psychometrics dataset; see the main text for details). Correlations in the whole sample (*r*) and partial correlations controlling for sex (*r*_p_) are displayed above the diagonal. The shaded areas in the scatterplots represent discordant profiles that are male-directional but female-typical, or vice versa. M-F_D_ = sex-directionality; M-F_T_ = sex-typicality; M-F_P_ = sex-probability; M-F_C_ = sex-centrality

The last example I present in this section comes from a large online study of personality carried out by the Open-Source Psychometrics Project (https://openpsychometrics.org). In this study, the 15 primary personality factors of Cattell’s 16PF model were measured using public domain items. Figure 14 is based on the US subsample of the dataset (7974 males and 13,607 females, 16–90 years), which was previously analyzed by Kaiser et al. (2020). The Mahalanobis distance between the male and female centroids was *D*_M_ = 1.17 (*D*_Mu_ = 1.17), and the proportion of discordant profiles was 12.6%. With 15 traits, the data are high-dimensional enough that most of the probability mass is concentrated in the outer regions of the distribution (see Del Giudice, 2023a). Thus, one should keep in mind that even highly sex-central profiles are unlikely to lie in the vicinity of the corresponding centroid.

Overall, the four indices were more strongly correlated than in the other datasets, both in the whole sample and within each sex. The partial correlation between M-F_D_ and M-F_T_ scores was rather high, with *r*_p_ = .88. Most likely, this was due to the comparatively high levels of measurement error in personality scores; Kaiser et al. (2020) estimated the reliability of the 15 scales with Cronbach’s *α*; values ranged from .68 to .91, with an average of *α =* .83.

**Fig. 15:**
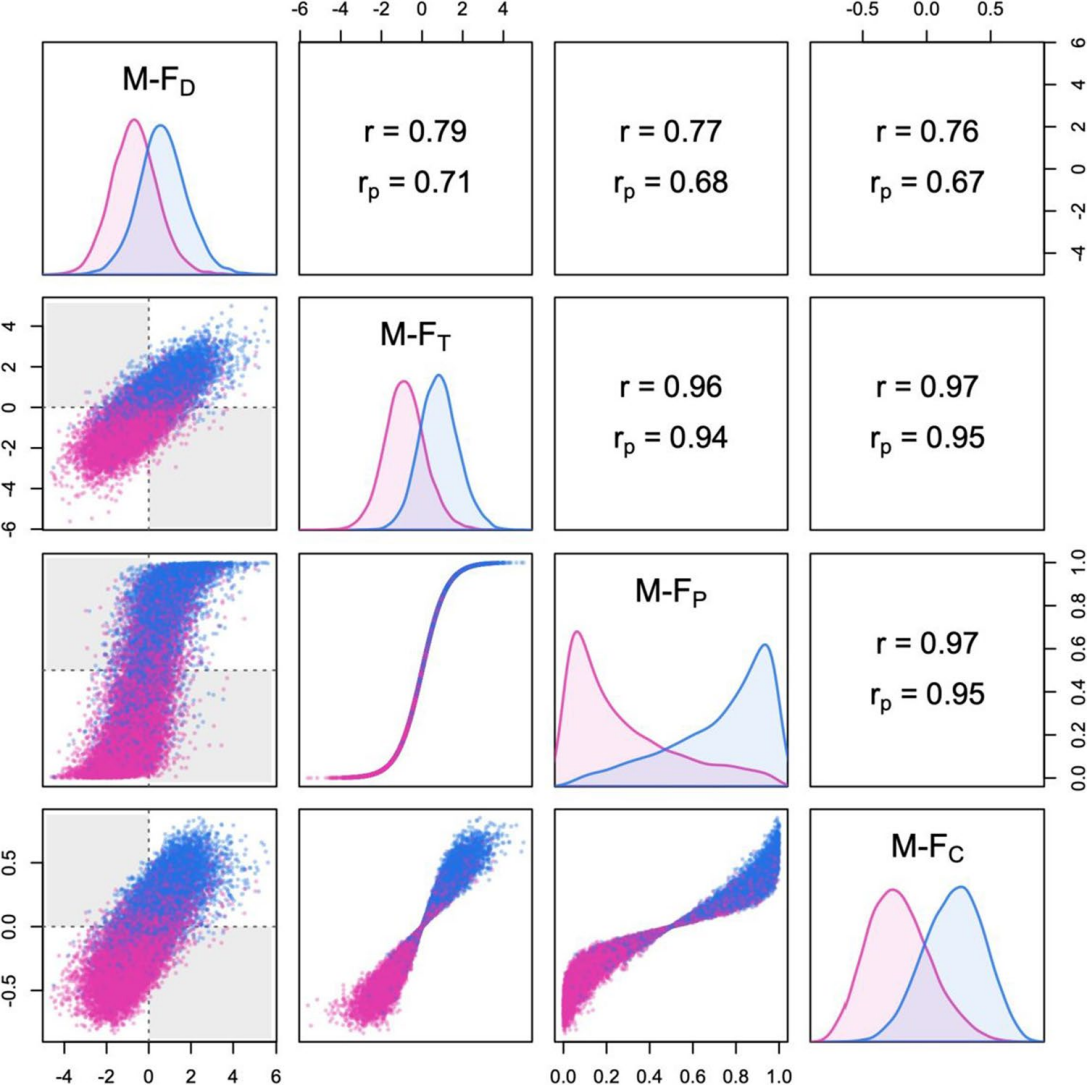
Summary plot of M-F indices measuring masculinity-femininity in personality traits (see Fig. 14), after measurement error correction with the DMD method. Correlations in the whole sample (*r*) and partial correlations controlling for sex (*r*_p_) are displayed above the diagonal. The shaded areas in the scatterplots represent discordant profiles that are male-directional but female-typical, or vice versa. M-F_D_ = sex-directionality; M-F_T_ = sex-typicality; M-F_P_ = sex-probability; M-F_C_ = sex-centrality

Together with the large sample size, the fact that reliability estimates for the personality scores are readily available makes this dataset an ideal candidate to attempt error correction with DMD. The corrected results are shown in Fig. 15. After correction, the Mahalanobis distance between centroids rose to *D*_M_ = 1.67 (*D*_Mu_ = 1.67), with 18.3% of profiles classified as discordant. The increased separation between the male and female distributions was underscored by a more skewed distribution of M-F_P_ scores. At the same time, the partial correlation between M-F_D_ and M-F_T_ scores decreased to *r*_p_ = .71; as expected, adjusting trait correlations to counteract the attenuating effect of measurement error revealed a clearer distinction between sex-typicality and sex-directionality than initially suggested by the raw data. The M-F scores calculated from the corrected data are also expected to be noticeably more valid than those calculated from the observed data, especially in the case of M-F_T_, M-F_P_, and M-F_C_ (see the online supplement).

## Conclusion

Various kinds of statistical M-F indices have been used for almost 100 years, but a systematic presentation of their characteristics and mutual relations has been lacking. In this paper, I laid out a theoretical and practical framework for the multivariate assessment of masculinity-femininity. I also discussed some important issues that had not been adequately addressed in the earlier literature, such as the emergence of reversals and discordant profiles and the profound impact of measurement error on the validity of M-F indices. I hope this synthesis will help bring conceptual clarity to the field, and encourage researchers to probe the usefulness of alternative indices in a variety of research contexts. Despite many decades of debate and study, the interface between sex and individual differences is still largely uncharted. While methodology quickly becomes sterile without the guide of theory, it is also true that theoretical progress is often aided by advances in measurement. Sex/gender research is no different; we should constantly strive to refine our tools and develop a clear, sophisticated understanding of how they work.

### Supplementary information

Below is the link to the electronic supplementary material.

Supplementary file1 (PDF 1383 KB)

## Data Availability

The data (when available for sharing) for the analyses reported in the manuscript are available at 10.6084/m9.figshare.22277758

## Footnotes

1 In a recent chapter on the measurement of sex differences (Del Giudice, 2022), I referred to the centroid axis as the “M-F axis”; but this was a poor terminological choice because it invites confusion between the general idea of an M-F continuum and a specific way of conceptualizing and measuring it.

2 This weighting scheme implies that the resulting M-F_D_ scores will be dominated by the traits showing the largest univariate differences. An alternative approach not explored here (but common in the early days of M-F research) is to compute an unweighted sum of traits (e.g., by replacing Cohen’s *d* values in vector **d** of Eq. 1 with +1 or –1, depending on the direction of the sex difference on each trait). Such unweighted sex-directionality scores may be regarded as more “balanced” because they give the same importance to all the traits, and are more robust to sampling error (e.g., Dawes, 1979). In practice, these two kinds of sex-directionality scores tend to converge when sex differences are relatively homogeneous across traits and/or the set of traits is sufficiently large.

3 The Mahalanobis distance ${D}_{{\text{M}}}({\mathbf{z}}_{1},{\mathbf{z}}_{2})=\sqrt{{({\mathbf{z}}_{1}-{\mathbf{z}}_{2})}^{{\text{T}}}{\mathbf{R}}^{-1}({\mathbf{z}}_{1}-{\mathbf{z}}_{2})}$ is a generalization of the standardized Euclidean distance that takes correlations into account (see Del Giudice, 2023a; Huberty, 2005). Specifically, *D*_M_ corresponds to length of the straight-line segment between two points, divided by the value of the multivariate *SD* along the direction of that segment. In the formula, **z**_1_ and **z**_2_ are the vectors of standardized scores corresponding to the two points, and **R** is the correlation matrix (pooled in the case of two groups). If the traits are all orthogonal, the correlation matrix becomes the identity matrix and the Mahalanobis distance reduces to the standardized Euclidean distance *D*_2_.

4 In Del Giudice (2022), I defined sex-typicality as the non-orthogonal projection of individual profiles on the centroid axis in the direction of the classification boundary (instead of their orthogonal projection on the discriminant axis, as described here). This was intended as a way to simplify the explanation by not having to explicitly discuss the difference between the centroid and discriminant axes; in retrospect, it made the explanation needlessly confusing even if technically true.

5 In practice, the difference between LDA and logistic regression tends to be negligible if the data do not deviate too dramatically from multivariate normality. I have compared the two methods on a variety of real-world psychological and anatomical datasets, and in every case the resulting scores have been almost perfectly correlated with each other (*r* > .99, typically *r* > .999).

6 In contrast to LDA and logistic regression, the probabilities generated by random forests, SVMs, and many other classifiers used in machine learning are generally not *calibrated*—that is, they do not correspond to the true relative frequency of the corresponding events, and hence cannot be interpreted in a frequentist sense. There are ways to transform the output of these classifiers into well-calibrated probabilities (see Niculescu-Mizil & Caruana, 2005).

7 Because of sampling error, estimates of ${D}_{{\text{M}}}$ can be substantially biased upward, especially when sample size is small relative to the number of traits. To address this problem, one can use the bias-corrected estimator ${D}_{{\text{Mu}}}=\sqrt{max\left[0,\left(\frac{{N}_{{\text{m}}}+{N}_{{\text{f}}}-k-3}{{N}_{{\text{m}}}+{N}_{{\text{f}}}-2}{D}_{{\text{M}}}^{2}-k\frac{{N}_{{\text{m}}}+{N}_{{\text{f}}}}{{N}_{{\text{m}}}{N}_{{\text{f}}}}\right)\right]}$ , where *k* is the number of traits, and ${N}_{{\text{m}}}$ and ${N}_{{\text{f}}}$ are the sizes of the male and female subsamples (see Del Giudice, 2022).

## Code availability

The code used for the analyses reported in the manuscript is available at 10.6084/m9.figshare.22277758

## Declarations

### Open practices statement

The data (when available for sharing) and code for all the analyses reported in the manuscript are available at 10.6084/m9.figshare.22277758
