# Supplementary material for: Statistical indices of masculinity-femininity: A theoretical and practical framework
Source: Behav Res Methods. 2024 Mar 4;56(7):6538–56. doi: 10.3758/s13428-024-02369-5 (PMC11362193; doi:10.3758/s13428-024-02369-5)
Supplement: Supplementary file 1 — Supplementary file1 (PDF 1383 KB) [file 13428_2024_2369_MOESM1_ESM.pdf]

Supplementary material for

Statistical Indices of Masculinity-Femininity: A Theoretical and Practical Framework

Marco Del Giudice

The four M-F indices discussed in the main text were calculated using LDA in simulated samples (multivariate normal) with *N* = 1,000 in each sex (total *N* = 2,000). Four levels of trait reliability (0.50, 0.70, 0.90, and 0.99) were considered, and simulations were repeated with different numbers of traits (*k* = 5, 10, and 30). Correlation matrices were generated with the vine method (Lewandowski et al., 2009), keeping the beta parameter fixed at 4. Each boxplot in the figures summarizes the distribution of results across 100 simulated samples. In the simulations presented in this supplement, the data were subjected to error correction with Data Matrix Disattenuation (DMD; Del Giudice, 2023) prior to calculating M-F indices. Disattenuation was based on the true (population) trait reliability; note that the DMD method performs very similarly when reliabilities are estimated with some error rather than accurately known (see Del Giudice, 2023).

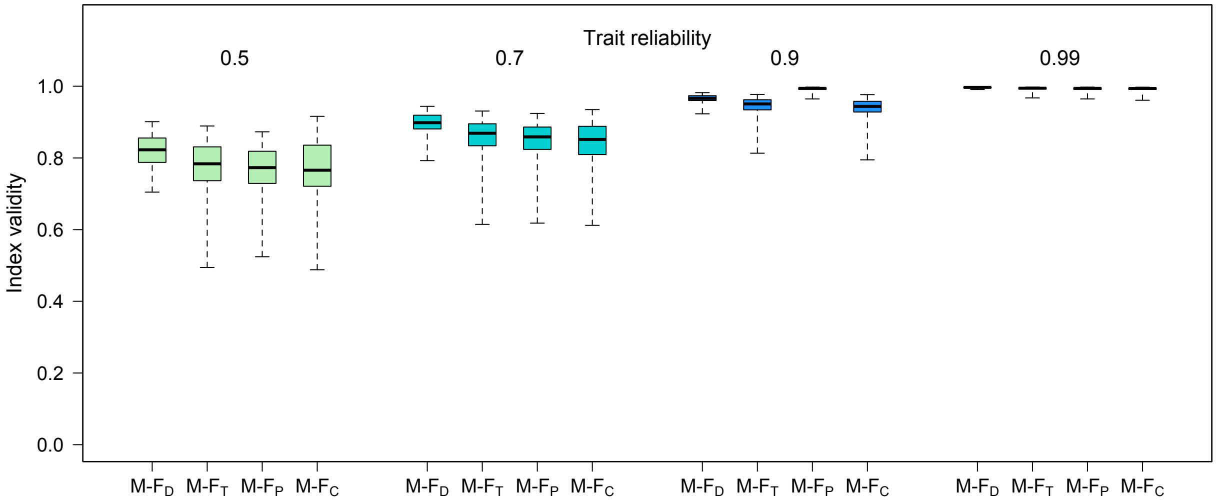

Fig. S1. Validity of the four M-F indices after DMD correction for measurement error, at different levels of trait reliability with *k* = 5 traits. The mean absolute true correlations between traits were in the .20-.25 range; univariate sex differences (Cohen's *d*) were normally distributed with mean 0 and SD = 0.70, and the true Mahalanobis distance between males and females had an average of about 2.

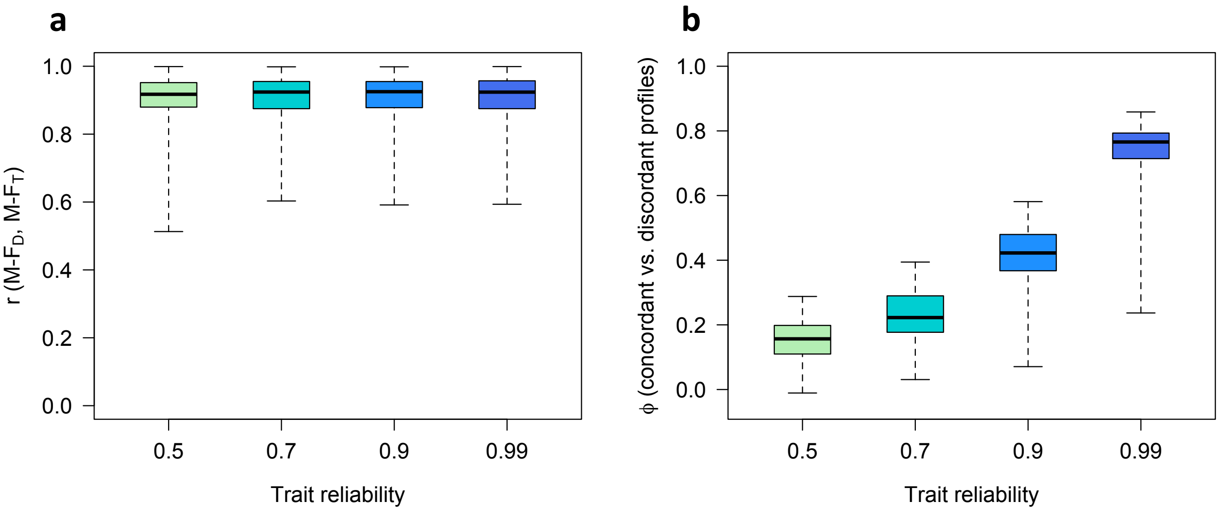

Fig. S2. Panel (**a**): observed correlation between sex-directionality (M-F$_D$) and sex-typicality (M-F$_T$) after DMD correction for measurement error, at different levels of trait reliability with *k* = 5 traits. a Panel (**b**): Phi coefficient for concordant vs. discordant profiles (i.e., profiles showing M-F$_D$ and M-F$_T$ scores with the same or opposite signs) after DMD correction for measurement error, at different levels of trait reliability with *k* = 5 traits. All simulation parameters were the same as in Fig. S1.

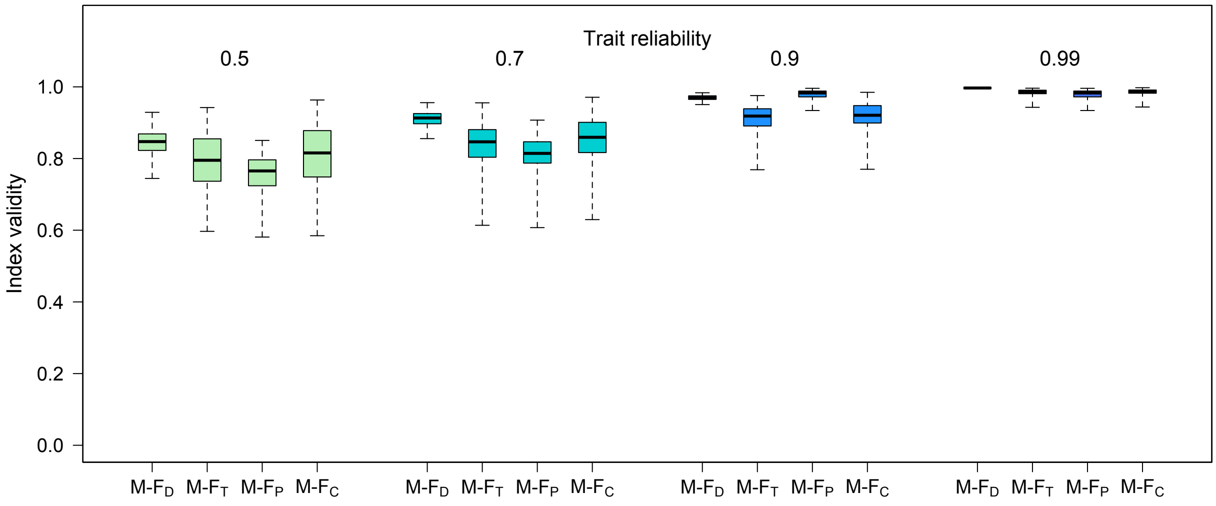

Fig. S3. Validity of the four M-F indices after DMD correction for measurement error, at different levels of trait reliability with *k* = 10 traits. The mean absolute true correlations between traits were in the .20-.25 range; univariate sex differences (Cohen's *d*) were normally distributed with mean 0 and SD = 0.50, and the true Mahalanobis distance between males and females had an average of about 3.

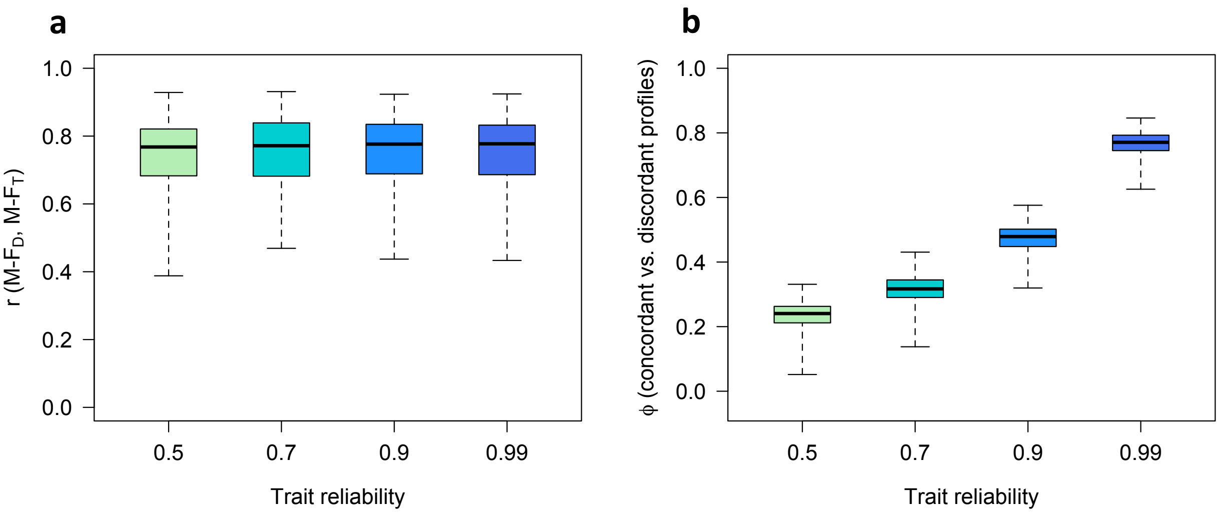

Fig. S4. Panel (**a**): observed correlation between sex-directionality (M-F$_D$) and sex-typicality (M-F$_T$) after DMD correction for measurement error, at different levels of trait reliability with *k* = 10 traits. Panel (**b**): Phi coefficient for concordant vs. discordant profiles (i.e., profiles showing M-F$_D$ and M-F$_T$ scores with the same or opposite signs) after DMD correction for measurement error, at different levels of trait reliability with *k* = 10 traits. All simulation parameters were the same as in Fig. S3.

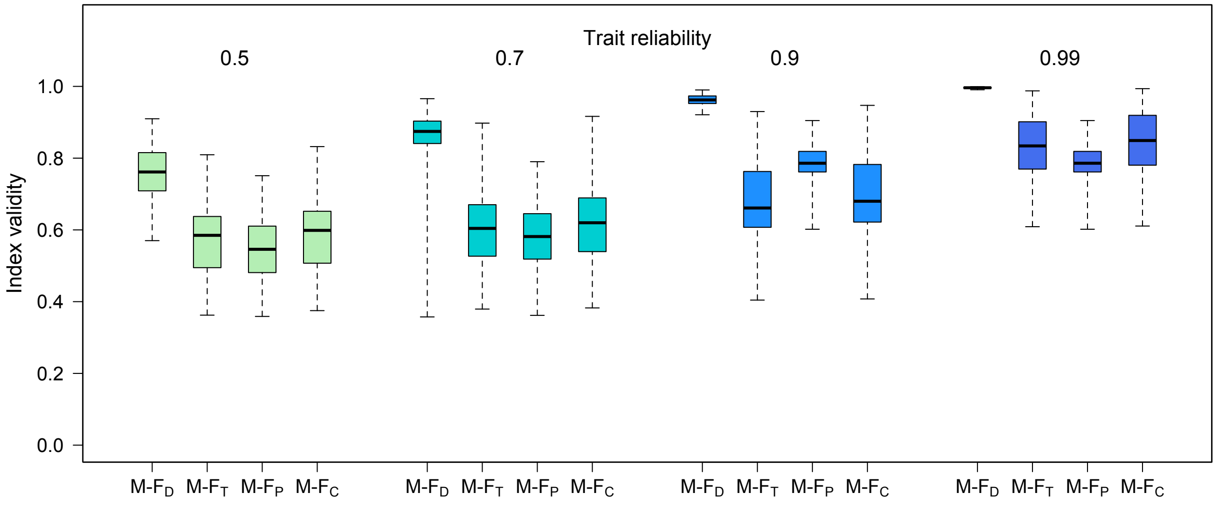

Fig. S5. Validity of the four M-F indices after DMD correction for measurement error, at different levels of trait reliability with k = 30 traits. The mean absolute true correlations between traits were in the .20-.25 range; univariate sex differences (Cohen's d) were normally distributed with mean 0 and SD = 0.05, and the true Mahalanobis distance between males and females had an average of about 4.

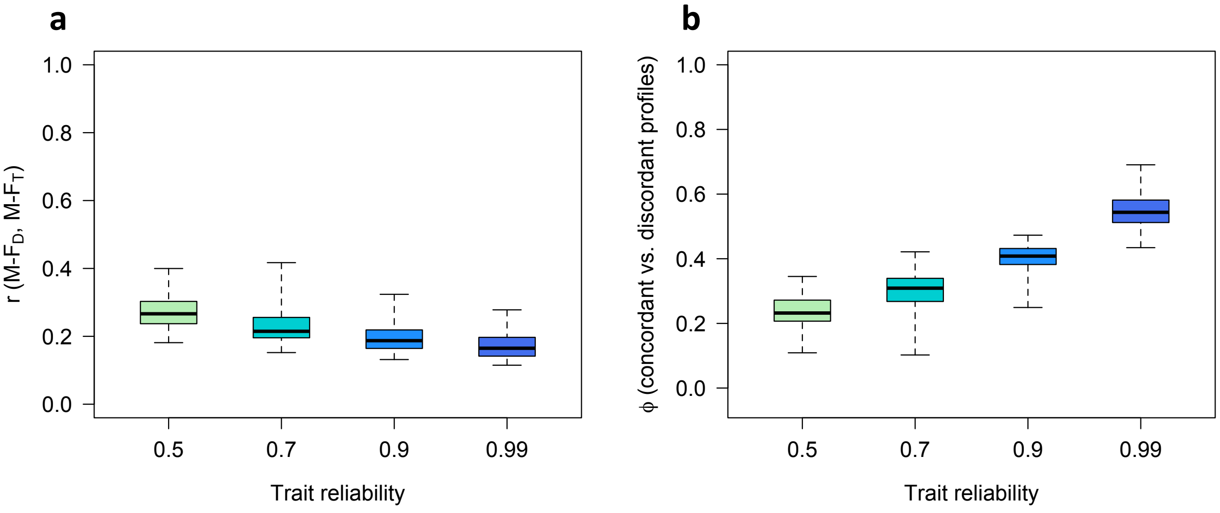

Fig. S6. Panel (**a**): observed correlation between sex-directionality ($M\text{-}F_D$) and sex-typicality ($M\text{-}F_T$) after DMD correction for measurement error, at different levels of trait reliability with k = 30 traits. Panel (**b**): Phi coefficient for concordant vs. discordant profiles (i.e., profiles showing $M\text{-}F_D$ and $M\text{-}F_T$ scores with the same or opposite signs) after DMD correction for measurement error, at different levels of trait reliability with k = 30 traits. All simulation parameters were the same as in Fig. S5.

References

Del Giudice, M. (2023). Data matrix disattenuation: A simple, effective method for correcting measurement error in multivariate datasets. PsyArXiv, https://doi.org/10.31234/osf.io/9kaw6

Lewandowski, D., Kurowicka, D., & Joe, H. (2009). Generating random correlation matrices based on vines and extended onion method. *Journal of multivariate analysis, 100,* 1989-2001. https://doi.org/10.1016/j.jmva.2009.04.008
